# Wnt5a gain- and loss-of-function in bone have distinct craniofacial phenotypes

**DOI:** 10.1093/jbmrpl/ziag060

**Published:** 2026-04-07

**Authors:** Claire J Houchen, Portia Hahn Leat, Cassandra Delich, Jocelyn Vang, Sara Yingling-Haggard, Joseph L Roberts, Hicham Drissi, Erin E Bumann

**Affiliations:** School of Dentistry, University of Missouri-Kansas City, Kansas City, MO 64108, United States; School of Dentistry, University of Missouri-Kansas City, Kansas City, MO 64108, United States; School of Dentistry, University of Missouri-Kansas City, Kansas City, MO 64108, United States; School of Dentistry, University of Missouri-Kansas City, Kansas City, MO 64108, United States; School of Dentistry, University of Missouri-Kansas City, Kansas City, MO 64108, United States; Emory School of Medicine, Emory University, Atlanta, GA 30322, United States; College of Health Solutions, Arizona State University, Phoenix, AZ 85004, United States; Emory School of Medicine, Emory University, Atlanta, GA 30322, United States; Atlanta VA Medical Center, Decatur, GA 30033, United States; School of Dentistry, University of Missouri-Kansas City, Kansas City, MO 64108, United States

**Keywords:** Robinow syndrome, craniofacial abnormalities, facial asymmetry, tooth eruption, micrognathism, mandibular condyle, apoptosis

## Abstract

Robinow syndrome has characteristic craniofacial and dental features and can be caused by gain- or loss-of-function variants in Wnt family member 5A (*WNT5A*) non-canonical signaling. The craniofacial and dental manifestation of Robinow syndrome is heterogenous, as is the effect of altered *Wnt5a* in animal models. The relationship between *Wnt5a* and craniofacial and dental phenotypes is not fully understood. To investigate the role of *Wnt5a* in bone during craniofacial and dental development, we utilized a *Wnt5a* conditional loss-of-function (LOF: *Wnt5a^fl/fl^;Ctsk^cre^*) and a *Wnt5a* conditional gain-of-function (GOF: *Rosa26-LSL-Wnt5a;Ctsk^cre^*) model to determine the effect of both LOF and GOF of *Wnt5a* in bone cells during craniofacial and dental development. Postnatal day 10 conditional LOF *Wnt5a*, GOF *Wnt5a*, and control skulls were scanned by micro-CT and assessed using traditional and geometric morphometrics. Mandibular bone apoptosis was further assessed by TUNEL staining. Conditional *Wnt5a* LOF resulted in midface hypoplasia, increased maxillary intermolar width, increased rostral basisphenoid width, and delayed molar eruption. *Wnt5a* LOF mandibles did not have altered BMD or bone microarchitecture unlike our previous study examining *Wnt5a* LOF femurs. In contrast, conditional *Wnt5a* GOF results in macrocephaly, shortened hard palate, increased zygomatic length, micrognathia, and mandibular process dysmorphology. The micrognathia and mandibular process dysmorphology in the *Wnt5a* GOF mice were not due to increased apoptosis. A partially penetrant snout deviation was present in both the *Wnt5a* LOF and GOF mice. Craniofacial and dental phenotype differed between mice with conditional GOF and LOF of *Wnt5a*, consistent with the craniofacial phenotype heterogeneity in *WNT5A*-associated Robinow syndrome. We detected tooth eruption delay, mandibular condyle dysmorphology, and facial asymmetry in mice with altered *Wnt5a* that have not been previously reported in patients with *WNT5A*-associated Robinow syndrome. Our data suggest precise regulation of *Wnt5a* is essential for proper craniofacial and dental development.

## Graphical Abstract

**Graphical Abstract:**
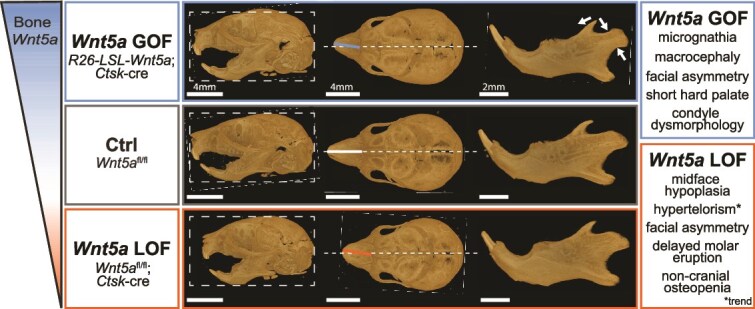

## Introduction

Craniofacial anomalies affecting the head, mouth, or jaw are common and impact 2%-3% of all babies.^1^ Craniofacial anomalies can be isolated and of non-syndromic origin or can occur as part of a syndrome. One such syndrome, a disorder called Robinow syndrome, has characteristic skeletal, craniofacial, and dental anomalies.^2^^,^^3^ Robinow syndrome occurs due to pathogenic variants in Wnt family member 5A (*WNT5A*) or other members of the *WNT5A* non-canonical signaling pathway, such as receptor tyrosine kinase-like orphan receptor 2, disheveled 1, disheveled 3, and nucleoredoxin.^4–6^ Robinow syndrome is the only congenital defect associated with *WNT5A.* Characteristic skeletal features include limb shortening and brachydactyly, but there is heterogeneity in the specific craniofacial manifestations of Robinow syndrome, even among individuals with mutations in the same gene. For example, the craniofacial phenotype of individuals with *WNT5A*-associated Robinow syndrome include midface hypoplasia, hypertelorism, macrocephaly, short hard palate, micrognathia, cleft lip/palate, delayed tooth eruption, and others, but not all pathogenic variants in *WNT5A* result in the same set of craniofacial anomalies.^6–8^ Unfortunately, the rarity and heterogeneity of Robinow syndrome makes affected individuals challenging to identify for referral for proper diagnostic testing.^6^ A greater understanding of how Robinow syndrome craniofacially and dentally manifests is critical for decreasing time to diagnosis and improving treatment for affected individuals.

Recent evidence suggests that perturbations in *WNT5A* non-canonical signaling from loss-of-function (LOF), gain-of-function (GOF), or hypomorphic variants can all result in Robinow syndrome.^7^^,^^9^^,^^10^ The relationship between variant effect and craniofacial phenotype is not fully understood, but animal models have begun to shed light on the role of *Wnt5a* in craniofacial and dental development.^11–15^ Severe skeletal defects including an extreme craniofacial phenotype can be observed in E18.5 *Wnt5a*^−/−^ embryos, but *Wnt5a* global KO is perinatal lethal in mice and is consequently neither usable for assessing postnatal craniofacial morphology nor likely representative of the Robinow syndrome patient population.^11^ Dentally, *Wnt5a* is known to influence molar cusp morphology and root development, and is robustly expressed in alveolar bone during tooth eruption; however, whether alterations in non-canonical Wnt signaling results in eruption changes is not fully understood.^8^^,^^13^^,^^15–17^ Delayed eruption has been reported in an individual with *DVL1*-associated Robinow syndrome.^8^ The mechanism by which *Wnt5a* impacts craniofacial and dental phenotype remains incompletely described.

**Figure 1:**
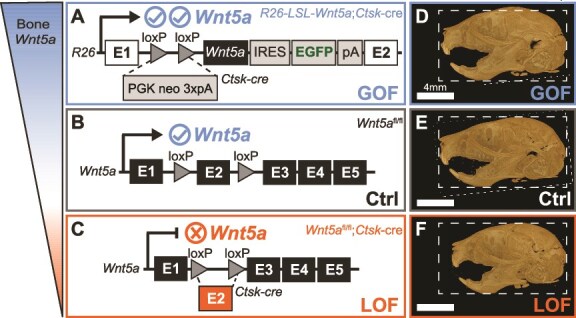
*Wnt5a* gain-of-function (GOF), control (Crtl), and loss-of-function (LOF) mice. (A) Conditional *Wnt5a* GOF (*Rosa26-LSL-Wnt5a;Ctsk^cre^*) mice,^35^ (B) control *Wnt5a* floxed mice (*Wnt5a^fl/fl^*), and (C) conditional *Wnt5a* LOF (*Wnt5a^fl/fl^;Ctsk^cre^*) genotype^31^ descriptions. Lateral micro-CTs of postnatal day 10 (D) *Wnt5a* GOF, (E) control, and (F) *Wnt5a* LOF mice demonstrate the craniofacial and dental phenotype of each group. Dashed line indicates control skull size.

Wnt5a has many known functions in bone tissue. In mouse studies, loss of *Wnt5a* is associated with decreased bone formation and decreased bone resorption.^18^  *Wnt5a*-mediated signaling promotes differentiation of osteoblasts, cells that deposit bone, and promotes the formation of osteoclasts, cells that resorb bone.^18–20^ Both osteoblasts and osteoclasts have known roles in craniofacial development.^21–25^ However, the extent to which craniofacial phenotype is impacted by *Wnt5a* signaling within skeletal tissue specifically must be determined to elucidate the craniofacial locations in which *Wnt5a*-mediated signaling controls morphogenesis. The present study compares the craniofacial phenotype of mice with gain- or loss-of-function *Wnt5a* in bone cells and fibrocartilage, which does not include Meckel’s cartilage, using a cathepsin K cre (*Ctsk^cre^*) model to determine the extent to which craniofacial phenotypes differ (Figure 1A-C).

Originally the *Ctsk^cre^* model was designed to investigate bone-resorbing osteoclasts, but the cre recombinase is also expressed bone-depositing osteoblasts, osteocytes, bone lining cells in bone, and fibrocartilage, in addition to other organs, tissues, and cell types.^26^ In the mandible of *Ctsk^cre^* mice, *cre* expression is in osteoclasts, osteoblasts, periosteal cells, pericytes, bone marrow stromal cells, and the fibrocartilage in the temporomandibular joint (TMJ).^27^ Cathepsin K-positive cells express cre recombinase in the *Ctsk^cre^* model after embryonic day 14.5 when cathepsin K expression can first be detected.^28^ Cathepsin K-positive cells are essential for proper craniofacial and dental development, demonstrated by the craniofacial and dental changes in *Ctsk*^cre^;*DTA^fl/+^* mice.^25^ Cell types that influence craniofacial bone development and express *Wnt5a*, *Ctsk*, and are positive for cre recombination in *Ctsk^cre^* mice include osteoclasts,^26^^,^^29–31^ osteoblast lineage cells,^16^^,^^20^^,^^26^^,^^27^^,^^32^ and fibrocartilage.^27^^,^^33^^,^^34^

In summary of the existing literature, both *Ctsk^cre^*-expressing cells and *Wnt5a* independently are known to impact craniofacial and dental development. We sought to delineate which craniofacial features are affected by alterations in *Wnt5a* in bone tissue, as opposed to craniofacial features that may be altered in Wnt5a global KO mice and individuals with *WNT5A*-associated Robinow syndrome due to alterations in other tissues like Meckel’s cartilage. While the bone phenotype of *Wnt5a^fl/fl^;Ctsk^cre^* mice has been examined in the appendicular (femur) and axial (vertebral body) skeleton,^31^ the role of *Wnt5a* in *Ctsk^cre^*-expressing cells is not described in craniofacial bone. To investigate the role of bone *Wnt5a* in craniofacial and dental development in postnatal embryos, we utilized a *Wnt5a* conditional KO model (LOF: *Wnt5a^fl/fl^;Ctsk^cre^*) and a *Wnt5a* conditional overexpression model (GOF: *Rosa26-LSL-Wnt5a;Ctsk^cre^*) that allows us to investigate both LOF and GOF *Wnt5a* effects in bone cells on craniofacial development (Figure 1A-C). We hypothesized craniofacial phenotypes of conditional *Wnt5a* GOF mice would significantly differ compared to controls, and that craniofacial phenotypes of conditional *Wnt5a* LOF mice would significantly differ compared to controls. Craniofacial phenotypes of interest include size, BMD, bone microarchitecture, shape variation, and histological features. Outside of the Robinow syndrome patient population, this mouse study demonstrates the important skeletal, craniofacial, and dental role of *Wnt5a*.

## Materials and methods

### Animals

*Ctsk^cre^* mice were acquired from the University of Tokyo^29^ and have been backcrossed into a C57BL/6J background. *Wnt5a^fl/fl^* (Strain #: 026626) mice were purchased from Jackson Laboratory. Conditional *Wnt5a* KO (LOF) mice were generated as previously reported (Figure 1B and C).^31^  *Rosa26-LSL-Wnt5a* mice were acquired from Washington University, where they were originally generated and backcrossed into the C57BL/6J background through >10 breeding cycles.^35^ Homozygous *Rosa26-LSL-Wnt5a* mice were crossed with heterozygous *Ctsk^cre^* to generate condition *Wnt5a* knock-in (GOF) mice (Figure 1A). *Wnt5a* LOF and *Wnt5a* GOF mice were compared to *Wnt5a^fl/fl^* controls. Six or more mice per genotype were analyzed to generate a sufficient sample size for craniofacial morphometrics while minimizing excess animal use. All described methods were conducted on mixed sex groups containing males and females. Sex differences were not analyzed as the sample size per genotype per sex did not sufficiently power statistical comparison between groups stratified by sex. The only inclusion criteria was life at postnatal day 10; no difference in pup viability was noted between groups.

Mice were maintained in accordance with applicable state and federal guidelines and all experimental procedures were approved by the Atlanta VA Medical Center Institutional Animal Care and Use Committee (Protocol number: V008-23). Mice were housed at the Atlanta VA Medical Center with controlled conditions (21-24 °C temperature; 40%-70% humidity; 12 h/12 h light/dark cycle) with free access to Teklad #2018S food and water (0.1 μm filtered).

### Micro-CT scanning, reconstruction, and landmark placement

Ethanol-fixed heads from postnatal day 10 (P10) control (*n* = 6), GOF (*n* = 8), and LOF (*n* = 6) mice and scanned using a SkyScan 1275 micro-CT system (Bruker). A custom 0.5 mm aluminum filter was used. Samples were scanned at a voltage of 55 kV and a current of 180 μA with an integration time of 45 ms and a resolution of 14 μm^3^. Samples were scanned in 180° at a rotation step of 0.3°.

Reconstruction was completed using NRecon software version 1.7.4.2 (Bruker). Gaussian smoothing, ring-artifact reduction, and beam-hardening correction were applied, and the same thresholding parameters were used for each sample. Three-dimensional rendering and landmark placement were performed using Drishti software version 2.6.5 (National Computational Infrastructure’s VizLab). Landmarks were positioned on the skulls and mandibles as previously published.^36–38^ All landmarks were placed by the same blinded observer and intraobserver analysis was conducted to ensure replicability, see the “Statistics” section.

### Traditional morphometrics using all landmarks

Euclidean measurements, including angles and linear distances, between landmark points were taken for all measurements. Projected measurements were found by calculating the distance between parallel planes intersecting landmark points, as previously described.^36^ The mean of right and left measurements was used to compare all bilaterally paired landmarks between groups. All results including significant and non-significant measurements are in Table S1. All measurements were compared between groups as true measurements and adjusted for cranial or mandibular centroid size in order to mitigate any differences in skull size. Centroid size of the crania and mandibles were calculated using the root centroid size equation, $\mathrm{RCS}=\sqrt{\sum_{i=1}^n{\left({x}_i-\overline{x}\right)}^2+{\left(y;-\overline{y}\right)}^2+{\left({z}_i-\overline{z}\right)}^2}.$^39^ Each measurement is then a ratio of the size of the cranium or mandible, and comparisons can be made between groups.^40^^,^^41^

Snout asymmetry was analyzed by measuring the angle of deviation between the mid-sagittal plane and the internasal suture. Landmarks were placed at the anterior point of the nasal bone intersecting with the mid-sagittal plane, the nasion, and the anterior point of the nasal bone. An angular measurement was taken with the nasion as the vertex. Mild asymmetry was assigned if the value fell outside the range of asymmetry in the control group but was less than twice the most asymmetrical control. Pronounced asymmetry was assigned if the value was greater than twice the most asymmetrical control.

### BMD and bone microarchitecture

BMD and microarchitecture parameters were determined for all *N* = 20 samples using CTAnalyzer software version 1.17.7.2 + (Bruker). Calibration was done using Skyscan 0.30 and 1.25 g/cm^3^ CaHA mouse-sized (2 mm diameter and 10 mm length) density phantoms (Bruker). Measurements were analyzed for a Region of Interest (ROI) including the full skull, and with the ROI selected for the mandible only. A density threshold of 36-255 Hounsfield units (HU) was applied to the full skull ROI, and a density threshold of 36-255 HU was applied to the mandible ROI. The ROI was shrink-wrapped, and black and white speckles of greater than 10 voxels were removed. Bone volume in the mandibular condyle was calculated in CTAn by creating an ROI to separate the condyle from the rest of the mandible. The ROI spanned from the anterior-most point between the coronoid process and the condyle to the anterior-most point between the condyle and the angular process. This ROI was applied to all mandibles and minor adjustments were made to ensure the ROI was consistently placed in each individual.

### Geometric morphometric analyses

Shape differences in the coronoid and condylar processes of all *N* = 20 mandibles were analyzed using geometric morphometrics. The left and right side of each mandible were analyzed in case asymmetries were noted. Lateral images of the right and left mandible were captured from the 3D reconstructions of the skull micro-CT scans, including the location of the 11 landmarks placed on the mandible in 3D as described in the geometric morphometrics analyses section of the main methods, which were used to estimate mandible centroid size. Images of the right side of the mandible were mirrored to allow direct mandibular process shape comparison of both sides. A blinded observer placed 59 semi-landmarks on each image using tpsDig2 (v2.32).^42^ Two landmarks were also placed on either side of a scale bar for quality control to ensure all landmarks were scaled equally. The full description of mandibular 3D-placed landmarks and semi-landmarks placement are in Figure S1.

Landmark coordinates were analyzed using the *Geomorph* R package.^43^ Generalized Procrustes analysis (GPA) using the *gpagen* function was used to align the landmark coordinates to adjust for differences in scale, orientation, and position.

Procrustes coordinates from a GPA using all 3D-placed and semi-landmarks were used to assess shape variation using a principal component analysis with the *gm.prcomp* function. Only the principal component axes that represented greater than 10% of the shape variation were used in the multivariate analysis of covariance (MANCOVA) models; using *lm.rrpp* function (type III sums of squares, 10 000 iterations). Genotype, sex, side (right/left), and individual, and all their interactions were used as categorical independent variables, and log-transformed mandible centroid size as the covariate. Variables that were not significant were removed from the final model. The final model included only genotype and mandible centroid size. A pairwise test was used to look at the effect of genotype on shape using the *pairwise* function, which measured the Euclidean distance between group means (10 000 iterations, 0.95 confidence). The interactions between log transformed mandible centroid size and each genotype were used to test for allometric relationships with shape. A pairwise test using the *pairwise* function measured the distance between slope vectors for each genotype to determine if any genotype had an allometric slope that significantly differed from the others (10 000 iterations, 0.95 confidence).

### Tissue sectioning, H&E staining, and TUNEL labeling

Formalin-fixed whole heads were decalcified in 10% EDTA (pH 7.2) for 10 d. Tissues were then embedded in paraffin blocks and were stored at 4 °C. Blocks were placed in a beaker and chilled in an ice box to maintain low temperature during sectioning, which prevented tissue breakage. Using a microtome, control and experimental mice head tissues were sectioned transversely and sagittally. Serial sections of mice head tissue were cut in 7 μm sections and placed on a distilled water bath at 42 °C. Mice head sections were transferred from distilled water bath to superfrost/plus microscope slides for processing and staining. To qualitatively and histologically compare mandibular condyle anatomy, H&E staining was performed on sagittal sections: 3× xylene for 5 min, ethanol rehydration, hematoxylin for 8 min, water rinse, 1% acid alcohol dip, water rinse, eosin for 30 s, water rinse, ethanol dehydration, 3× xylene for 2 min, and coverslipped with Permount. TUNEL labeling using the Click-iT Plus TUNEL Assay Alexa Fluor 647 (Invitrogen, C10619) was conducted using the manufacturer’s protocol on 7 μm formalin fixed paraffin embedded transverse sections of control and *Wnt5a* GOF mice. Four loci in 3 anatomical regions were assessed for TUNEL per individual: mandibular bone sans teeth near the mandibular incisor, mandibular bone sans teeth near the mandibular molars including angular process cartilage, and mandibular condyle bone and cartilage (*n* = 2 mice/genotype, 3 sections/mouse, and 4 loci/section). TUNEL-stained sections were mounted in DAPI (4′,6-diamidino-2-phenylindole) mounting media (Vector Laboratories, H-1200-10), imaged the following day to prevent signal loss on a Keyence BZ-X810 Epifluorescence High Content Imaging System (Keyence), and DAPI-positivity and TUNEL-positivity was quantified using FIJI ImageJ.^44^

### Statistics

Outcome measures were traditional craniofacial morphometrics (primary outcome), tooth emergence, mandibular BMD and bone microarchitecture, geometric morphometrics of the mandibular processes, and TUNEL-indicated DNA damage. All comparisons except geometric morphometrics were done in GraphPad Prism v10.4.1 (GraphPad). For the traditional craniofacial morphometrics, after assessing normality and similarity of variance to ensure appropriateness of a parametric test, groups were compared using an unpaired, parametric, 2-tailed *t*-test with Welch correction to evaluate statistical significance, with the False Discovery Rate FDR (Q) set to 5. *Wnt5a* GOF were compared to control mice and *Wnt5a* LOF were compared to control mice, but *Wnt5a* GOF and *Wnt5a* LOF mice were not compared to one another. A stringent *p*-value of <.01 was used to determine if the traditional craniofacial morphometrics measurement likely meaningfully differed between groups. For all other *t* tests, an unpaired, parametric, 2-tailed *t*-test and a typical *p*-value of <.05 was used. A 2-way ANOVA was used to compare TUNEL:DAPI ratio between the three mandibular loci and between genotypes and a typical *p*-value of <.05 was used. All measurements were reported using scatter plots with bars representing mean and SD. Cohen’s *d* effect size was used to describe magnitude of difference between groups. No animals were excluded from the analysis.

Sample sizes (*n* = 6-8 per genotype) were selected based on prior craniofacial morphometric studies and the expected magnitude of biologically meaningful differences. Using observed SDs from representative craniofacial measurements, power calculations indicate that the present study with an *n* of 6-8 per genotype has >80% power (α = .05, 2-tailed) to detect between-group differences corresponding to standardized effect sizes (Cohen’s *d*) of ≥1.0, consistent with the effect sizes observed across all measurements in this study. Although *n* = 6-8 per group is typical in comparable murine studies and the assumptions of unpaired 2-tailed *t*-tests were assessed prior to hypothesis testing and statistical interpretation, we acknowledge that modest group sizes limit statistical power.

Three randomly selected samples from each genotype (*N* = 9) were landmarked a second time by the same observer to ascertain traditional morphometrics reliability. Measurements were considered replicable when the coefficient of variation between the 2 landmark sets was less than 10%. Table S2 contains the intraobserver coefficient of variation for all 114 measurements.

To ascertain the reliability of the cusp apex counting methodology, all individuals were assessed twice and the 2 observations were compared using intraclass correlation coefficients (ICC) in IBM SPSS Statistics (Version 29). The appropriate model for this experimental design is a 2-way mixed-effects model, single rater type, absolute agreement definition.^45^ The cusp apex counting methodology ICC 95% CI was 0.909 to 0.963, indicating excellent method reliability.^45^

## Results

**Figure 2:**
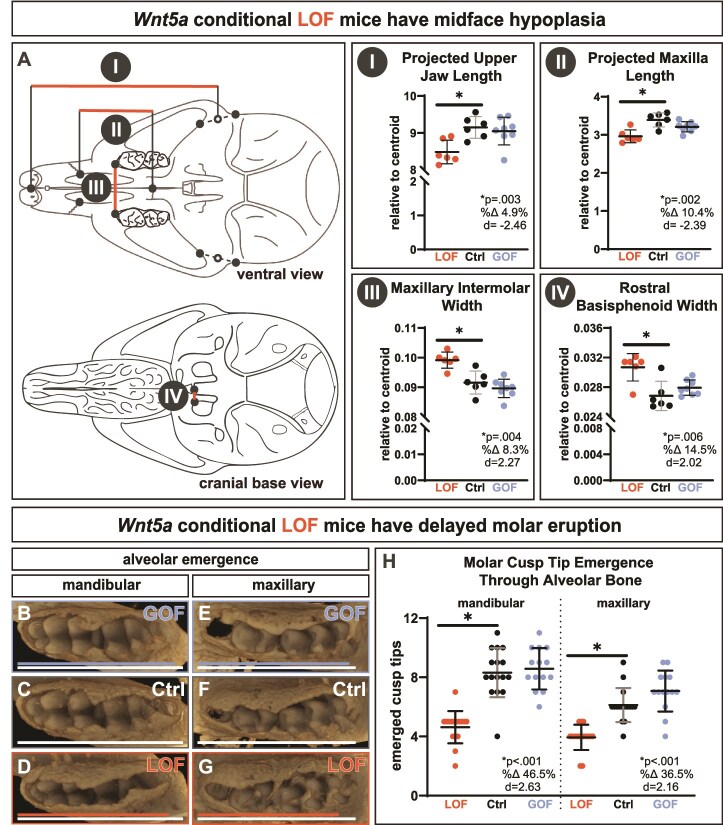
Conditional *Wnt5a* LOF mice have midface hypoplasia and delayed molar eruption. Determined by using anatomical landmarks on micro-CT scans, *Wnt5a* LOF mice alone had a significant (AI) decrease in projected upper jaw (maxilla + premaxilla) length, (AII) decrease in projected maxilla length, (AIII) increase in maxillary intermolar width, and (AIV) increase in rostral basisphenoid width in the cranial base. The occlusal view of the micro-CT scans demonstrate that in both (B-D) the mandibular molars and (E-G) the maxillary molars, bone covers or partially covers more cusp tips in P10 *Wnt5a* LOF mice. Solid lines on the occlusal view micro-CT scans indicate molar field length, which were decreased in the mandible and maxilla in *Wnt5a* LOF mice. (H) *Wnt5a* LOF mice have fewer mandibular and maxillary molar cusp tips completely uncovered by bone. Exact *p*-values, percent change, and Cohen’s *d* effect size are listed within each graph. The roman numeral label adjacent to the graph corresponds to the similarly labeled line in the schematic drawing.

*Wnt5a* LOF skull length relative to centroid size was a significant 1.7% shorter than controls (Figure 1D-F, Figures S2 and S7B). No body weight differences were detected at P10, indicating differences in body size are not the source of skull size changes (Figure S3). The trend toward shorter total skull length in *Wnt5a* LOF mice was due in part to midface hypoplasia (Figure 2A). Indicative of midface hypoplasia, *Wnt5a* LOF mice compared to controls had a significant 4.9% decrease in projected upper jaw (maxilla + premaxilla) length (Figure 2AI) and a significant 10.4% decrease in projected maxilla length (Figure 2AII). In the transverse dimension, *Wnt5a* LOF mice had a significant 8.3% larger maxillary intermolar width (Figure 2AIII) and a significant 14.5% wider rostral basisphenoid width than controls (Figure 2AIV). No difference in midface length or transverse cranial widths between *Wnt5a* GOF mice and controls was detected. Interorbital width trended 5.6% wider in *Wnt5a* LOF mice (Figure S4). Indicative of significant delayed molar eruption, *Wnt5a* LOF mice had 46.5% fewer mandibular (Figure 2B-D) and 36.5% fewer maxillary (Figure 2E-G) molar cusp tips fully unobstructed by alveolar bone at postnatal day 10 than controls (Figure 2H). Though severe molar morphology changes were not observed (Figure S5), *Wnt5a* LOF mice had a 6.5% decrease in mandibular molar field length and a 7.7% decrease in maxillary molar field length compared to controls indicating smaller teeth (Figure S6).

**Figure 3:**
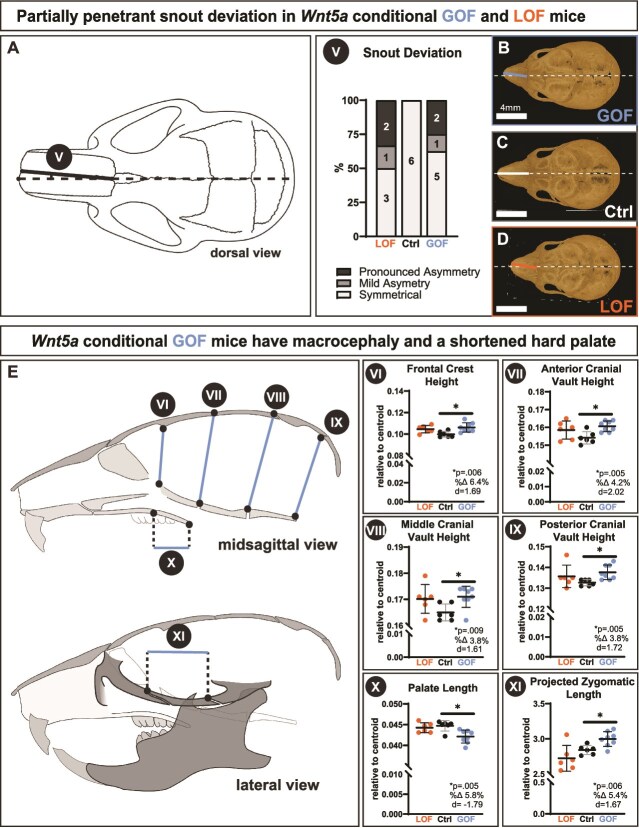
Both conditional *Wnt5a* LOF and GOF mice had a partially penetrant snout deviation, while GOF mice alone had macrocephaly and a shortened hard palate. (A) Approximately half of the *Wnt5a* LOF and GOF mice had a mild or pronounced snout deviation, indicative of facial asymmetry. (B-D) Dorsal view micro-CT images demonstrate the snout asymmetry, which deviated to the right in all affected individuals. *Wnt5a* GOF mice alone had a significant (EVI) increase in frontal crest height, (EVII) increase in anterior cranial vault height, (EVIII) increase in middle cranial vault height, (EIX) increase in posterior cranial vault height, which taken together indicate macrocephaly. *Wnt5a* GOF mice alone also had a significant (EX) decrease in palate length and (EXI) increase in projected zygomatic length. Exact *p*-values, percent change, and Cohen’s *d* effect size are listed within each graph. The roman numeral label adjacent to the graph corresponds to the similarly labeled line in the schematic drawing.

Both *Wnt5a* GOF and LOF genotypes had a partially penetrant snout deviation (Figure 3A). Half of the *Wnt5a* GOF mice had a snout deviation: 1/6 had mild asymmetry and 2/6 had a pronounced asymmetry (Figure 3AV, B, and C). Slightly less than half of the *Wnt5a* LOF mice had a snout deviation: 1/8 had mild asymmetry and 2/8 had a pronounced asymmetry (Figure 3AV, C, and D). All snout deviations in the asymmetrical Wnt5a GOF and LOF were toward the right. There were no observed overt changes in any cranial sutures (data not shown).

While the *Wnt5a* LOF mice had midface hypoplasia and delayed tooth eruption, *Wnt5a* GOF mice had macrocephaly, shortened hard palate, micrognathia, and mandibular process dysmorphology. Indicative of macrocephaly, *Wnt5a* GOF mice had a significant 6.4% larger frontal crest height (Figure 3VI), a significant 4.2% larger anterior cranial vault height (Figure 3VII), a significant 3.8% larger middle cranial vault height (Figure 3VIII), and a significant 3.8% larger posterior cranial vault height (Figure 3IX) compared to controls. In the anterior-posterior dimension, *Wnt5a* GOF mice had a significant 5.8% shorter palate length than controls (Figure 3X). However, *Wnt5a* GOF mice had a significant 5.4% longer projected zygomatic length than controls (Figure 3XI).

**Figure 4:**
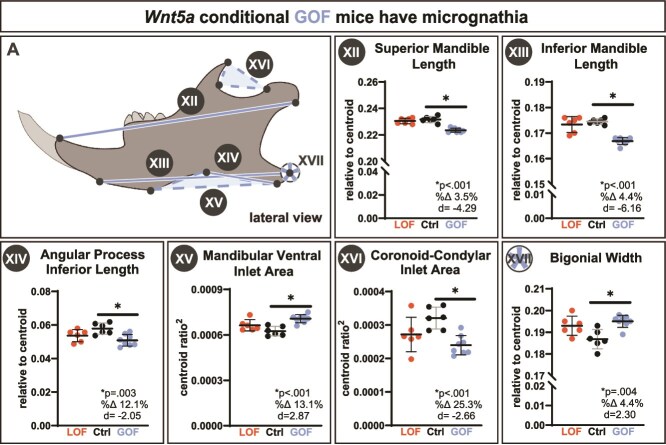
Conditional *Wnt5a* GOF mice had micrognathia, indicated by a significant decrease in (AXII) superior mandible length, (AXIII) inferior mandible length, and (AXIV) angular process inferior length. *Wnt5a* GOF mice also had changes in the mandibular processes, indicated by a significant (AXV) increase in mandibular ventral inlet area and (AXVI) decrease in coronoid-condylar inlet area. *Wnt5a* GOF mice had (AXVII) significantly wider bigonial width between the angular process of the left and right hemimandibles. Exact *p*-values, percent change, and Cohen’s d effect size are listed within each graph. The roman numeral label adjacent to the graph corresponds to the similarly labeled line in the schematic drawing.

Indicative of micrognathia, *Wnt5a* GOF mice had a significant 3.5% decrease in superior mandible length (Figure 4AXII) and a significant 4.4% decrease in inferior mandible length compared to controls (Figure 4AXIII). There were no biologically meaningful changes in cranial or mandibular centroid (Figure S7A and B) and BMD (Figure S7C and D). The only biologically meaningful bone microarchitecture change detected in the mandibles was a significant 11% decrease in bone surface (Figure S7E).

As determined by traditional morphometric analysis, *Wnt5a* GOF mice had a significant 12.1% smaller angular process inferior length (Figure 4AXIV), a significant 13.1% larger mandibular ventral inlet area (Figure 4AXV), and a significant 25.3% smaller mandibular coronoid-condylar inlet area than controls (Figure 4AXVI). The width between the left and right mandibular angular process (bigonial width) was significantly 4.4% wider in *Wnt5a* GOF mice (Figure 4AXVII). Notably, the *Wnt5a* GOF mice did not have a wider cranial base, indicating that the larger bigonial width in the *Wnt5a* GOF mice was not due to a widening of the cranial base.

**Figure 5:**
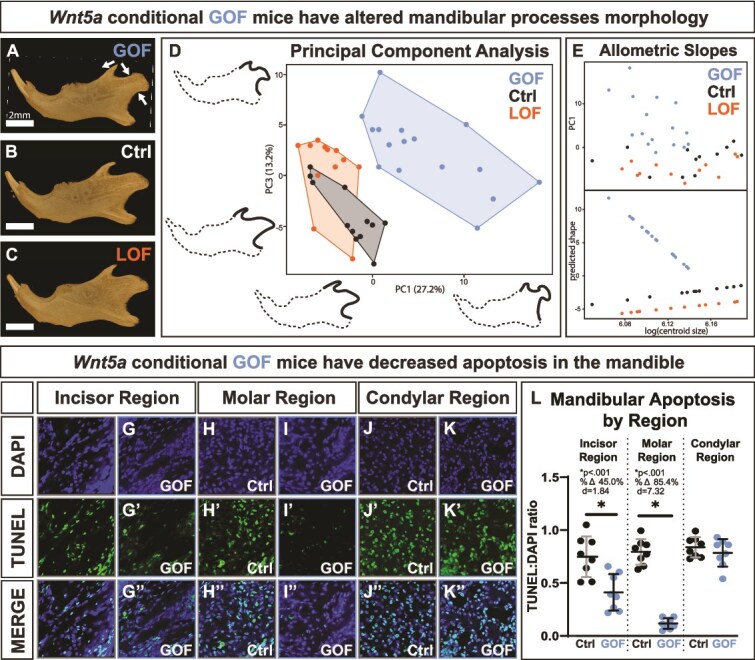
Conditional *Wnt5a* GOF mice had altered morphology of the mandibular processes as determined by geometric morphometrics but have decreased mandibular apoptosis. (A-C) Lateral view micro-CT images of segmented mandibles demonstrate the shorter and less rounded coronoid process, pinched ramus and rounded articular surface of the condylar process, and the downward tilted angular process in *Wnt5a* GOF mice. (D) PC1 (describes 27.2% of variation between samples) vs PC3 (describes 13.2% of variation between samples) of the principal component analysis of the mandibular process geometric morphometrics; outlines demonstrating phenotype are 2× exaggerated and to scale. (E) There is an allometric relationship between mandible size and mandibular process shape in the *Wnt5a* GOF mice alone. (F-K) Representative images of control and Wnt5a GOF mice stained for DAPI (blue) and TUNEL-indicated apoptosis (green). (L) The TUNEL:DAPI ratio was significantly decreased in *Wnt5a* GOF mice in mandibular tissue near the incisor and near the molars but was similar to controls in the mandibular condyle.

As determined by geometric morphometric analysis, there were morphological differences in the *Wnt5a* GOF coronoid and condylar processes (Figure 5A-C). Principal component analysis of GPA coordinates indicated principal components 1-4 each captured at least 10% of variation between samples. In *Wnt5a* GOF mice, coronoid processes tended to be shorter and less rounded, condylar processes tended to have a pinched ramus and a rounded articular surface, and the angular process tilted downward (Figure 5D). The full list of principal components are listed in Table S3, and the morphology captured by principal components 1-4 are visualized in Table S4. *Wnt5a* GOF mandibular process morphology differed significantly from both controls and *Wnt5a* LOF mice, and there was a significant allometric relationship between mandible centroid size and mandibular process shape in *Wnt5a* GOF mice only (Table S5). The allometric slope relating predicted shape to log mandible centroid size angles sharply downward in *Wnt5a* GOF mice alone, indicating that smaller mandible centroid size in *Wnt5a* GOF mice is associated with a more pronounced deviation in mandibular process shape (Figure 5E). There was no difference in mandibular condyle bone volume between genotypes, suggesting the *Wnt5a* GOF condyle phenotype is a matter of bone dysmorphology rather than underdevelopment (Figure S8).

Indicated by TUNEL staining (Figure 5F-K), *Wnt5a* GOF mice had a significant 45% decrease in apoptosis in mandibular bone near the incisor and an 85% decrease in apoptosis in mandibular bone near the molars compared to controls (Figure 5L). *Wnt5a* GOF mice had similar levels of TUNEL staining to controls in the mandibular condylar process.

## Discussion

One key finding of this study was that conditional LOF and conditional GOF of *Wnt5a* in mice results in distinct craniofacial phenotypes reminiscent of the heterogeneity of craniofacial phenotypes in individuals with Robinow syndrome. Craniofacial anomalies noted in patients with Robinow syndrome that were identified in this mouse study included midface hypoplasia and a trend toward hypertelorism only in the *Wnt5a* LOF group, while macrocephaly, shortened hard palate, and micrognathia were noted only in the *Wnt5a* GOF group (key findings are summarized in Table S6). These craniofacial anomalies are all associated with Robinow syndrome, but do not necessarily all co-occur in the same individuals.^6^ Our finding of a function-specific effect of *Wnt5a* is supported by other *Wnt5a* mouse models. For example, conditional *Wnt5a* LOF but not GOF in *Cdx2^cre^*, which targets posterior body structures, such as the hindlimbs and tail during embryonic development, mice results in severe hindlimb and tail truncation.^46^ While we predicted the craniofacial phenotype of *Wnt5a* LOF and GOF mice would differ from controls in many similar ways, they largely had completely distinct craniofacial changes compared to controls.

Another key finding of this study was the identification of several craniofacial and dental phenotypes not currently clinically associated with *WNT5A* mutations: tooth eruption delay, TMJ changes, and facial asymmetry. Delayed molar eruption was noted only in the *Wnt5a* LOF group, while mandibular process dysmorphology was noted only in the *Wnt5a* GOF group. Temporomandibular joint anomalies in individuals with Robinow syndrome are not reported in the literature, though there is an association between *Wnt5a* signaling and TMJ dysfunction in other animal models.^47^ Facial asymmetries were detected in both the *Wnt5a* LOF and GOF mice groups. Craniofacial asymmetries have not been reported in individuals with *WNT5A*-associated Robinow syndrome, but clinical photographs of individuals with Robinow syndrome suggest mild asymmetries may be present but not documented.^6^ WNT5A is involved in planar cell polarity and is needed for proper left-right patterning in early embryonic development, which may explain why some *Wnt5a* LOF and GOF mice displayed asymmetries in the midface and should be explored further.^12^^,^^48^ Delayed molar eruption, mandibular process morphology, and craniofacial asymmetries should be assessed in patients with *WNT5A*-associated Robinow syndrome. Cleft lip with/without cleft palate occurs in approximately 40%-60% of individuals with Robinow syndrome,^6^^,^^7^^,^^9^ but we did not detect clefting in this animal model nor did we detect a difference in perinatal lethality between genotypes.

Our findings are consistent with previous reports that *Wnt5a* GOF results in a shorter lower jaw.^12^ Viral overexpression of WT *WNT5A* and *WNT5A* with a missense variant in chick lower jaw results in a 20% reduction in lower jaw length.^12^ Conditional *Wnt5a* GOF, viral *Wnt5a* overexpression,^12^ viral missense variant-containing *Wnt5a* overexpression,^12^ and global *Wnt5a* KO^11^ cause micrognathia, but conditional *Wnt5a* LOF does not. This suggests *Wnt5a* LOF may be inconsequential in *Ctsk*-expressing periosteal cells, osteoclasts, pericytes, bone marrow stromal cells, and fibrocartilage in establishing mandibular size and shape, but essential in other cell types during jaw development. We found GOF but not LOF of *Wnt5a* in the aforementioned cell types disrupts critical processes required for jaw formation. Consequently, mandibular manifestations of *WNT5A* mutations are variant consequence-dependent, which may explain why micrognathia only occurs in 33%-57% of individuals with Robinow syndrome.^5^^,^^6^ Meckel’s cartilage, which is made of hyaline cartilage, is not positive for cre recombination in *Ctsk*-cre mice.^27^ Consequently, the micrognathia in the *Wnt5a* GOF mice is likely not due to undergrowth of the Meckel’s cartilage.

Global Wnt5a KO^13^ and *Ctsk*^cre^;*DTA*^*fl/+*25^ mice have altered molar morphology, but our conditional *Wnt5a* LOF and GOF mice did not have overtly altered molar morphology. This suggests *Wnt5a* affects tooth morphology, but not in *Ctsk* lineage cells. Conversely, *Ctsk* lineage cells affect tooth morphology via *Wnt5a-*independent mechanisms. Though we did not observe tooth morphology changes, we detected a trend toward decreased mandibular and maxillary molar field length in *Wnt5a* LOF mice that would not meet the clinical criteria for microdontia, and we detected delayed molar eruption in conditional *Wnt5a* LOF mice. *Wnt5a* is known to be robustly expressed in alveolar bone during tooth eruption and promote osteoclastogenesis,^16^^,^^20^ and our data provide a direct link between bone *Wnt5a* ablation and delayed tooth eruption.

Interestingly, the phenotype of our conditional *Wnt5a* GOF mice closely resembled that of *Ctsk*^cre^;*DTA^fl/+^* mice, with both exhibiting shortened mandibles, altered mandibular condyles, and enlarged cranial vaults.^25^ We consequently hypothesized that excess *Wnt5a* results in cell death in affected cells, explaining why loss of *Ctsk^cre^*-expressing cells and *Wnt5a* GOF in *Ctsk^cre^*-expressing cells results in a similar craniofacial phenotype. Unexpectedly, *Wnt5a* GOF mice had significantly decreased cell death in mandibular tissue. *Wnt5a* has a well-established association with decreased cell death, including in craniofacial tissue.^49–51^ It is possible physiological cell death during jaw development was disrupted in *Wnt5a* GOF mice resulting in craniofacial changes. Additionally, the *Ctsk*^cre^;*DTA^fl/+^* study and our own investigated slightly different cell populations, as the *Ctsk*^cre^;*DTA^fl/+^* study utilized a different *Ctsk*^cre^ mouseline.^30^ Further investigation using additional techniques to assess cell death is needed to definitively establish a connection between these phenomena, and to determine if structures that increased in size in *Wnt5a* GOF mice have less cell death.

Previously published data show male *Wnt5a* LOF mice have 38% lower femur BMD than controls,^31^ but interestingly, we detected no biologically meaningful difference in mandible BMD between groups (Figure S7C). This may explain why some individuals with *WNT5A*-associated Robinow syndrome have mild osteopenia of the non-cranial skeleton but normal BMD in the cranial skeleton.^52^ Bone surface was significantly decreased in *Wnt5a* GOF mandibles, but did not differ from controls in *Wnt5a* LOF mandibles in agreement with no reported difference in these measures in *Wnt5a* LOF femurs.^31^ However, other bone microarchitecture parameters were altered in the femurs of P70 male *Wnt5a* LOF mice but did not appear to differ between genotypes in the mandible at P10.^31^ To further assess a potential bone-specific difference, femur and mandible bone microarchitecture would need to be compared at P70. Similarly, *Wnt5a^fl/fl^;Osx^cre^* mice have decreased trabecular separation in the femur, but data in the mandible in this model is not available.^18^ The contrasting BMD and bone microarchitecture phenotypes between *Wnt5a* LOF femurs and mandibles indicate a potential bone-specific effect of *Wnt5a* LOF in *Ctsk^cre^*-expressing cells. Greater understanding of the bone cell types whose behavior is affected by *Wnt5a* will be gained by future studies using additional cre drivers, such as *LysM*-cre and *Osx*-cre, and the intracellular mechanism through which *Wnt5a* affects bone cell behavior will be investigated using these models.

In conclusion, *Wnt5a* alterations in bone were sufficient to alter craniofacial phenotype and both LOF and GOF of bone *Wnt5a* resulted in craniofacial changes. The craniofacial and dental phenotype differed between mice with conditional GOF and LOF of *Wnt5a* in *Ctsk^cre^*-expressing cells, consistent with other preclinical reports and the craniofacial phenotype heterogeneity in *WNT5A*-associated Robinow syndrome. Further, we detected TMJ changes and facial asymmetry in mice with altered *Wnt5a* that have not been previously reported but for which patients with Robinow syndrome should be monitored. We also detected tooth eruption delay in mice with altered *Wnt5a* which should be monitored for in individuals with *WNT5A*-associated Robinow syndrome. Lastly, *Wnt5a* may play a bone-specific role as demonstrated by differences in mandibles and femurs of conditional *Wnt5a* KO and in patients with Robinow syndrome. Our data suggest carefully regulated *Wnt5a* signaling within bone tissue is necessary for craniofacial development and that Robinow syndrome-associated craniofacial anomalies may arise in part due to alterations in bone.

## Supplementary Material

Houchen_Wnt5a_Supplement-PUBLICATION_CJH_033026_v11_ziag060

## Data Availability

Data not contained in the manuscript is available from the corresponding author on reasonable request.

## References

[1] Mossey PA, Catilla EE, eds. Global Registry and Database on Craniofacial Anomalies: Report of a WHO Registry Meeting on Craniofacial Anomalies. Geneva, Switzerland: World Health Organization; 2003.

[2] Bain MD, Winter RM, Burn J. Robinow syndrome without mesomelic 'brachymelia': a report of five cases. J Med Genet. 1986;23(4):350-354. 10.1136/jmg.23.4.350 3746837 PMC1049704

[3] Wadlington WB, Tucker VL, Schimke RN. Mesomelic dwarfism with hemivertebrae and small genitalia (the Robinow syndrome). Am J Dis Child. 1973;126(2):202-205. 10.1001/archpedi.1973.02110190176013 4724117

[4] Roifman M, Marcelis CLM, Paton T, et al. De novo-associated autosomal dominant Robinow syndrome suggests specificity of genotype and phenotype. Clin Genet. 2015;87(1):34-41. 10.1111/cge.12401 24716670

[5] Person AD, Beiraghi S, Sieben CM, et al. WNT5A mutations in patients with autosomal dominant Robinow syndrome. Dev Dyn. 2010;239(1):327-337. 10.1002/dvdy.22156 19918918 PMC4059519

[6] Conlon CJ, Abu-Ghname A, Raghuram AC, et al. Craniofacial phenotypes associated with Robinow syndrome. Am J Med Genet A. 2021;185(12):3606-3612. 10.1002/ajmg.a.61986 33237614 PMC13285961

[7] White JJ, Mazzeu JF, Coban-Akdemir Z, et al. WNT signaling perturbations underlie the genetic heterogeneity of Robinow syndrome. Am J Hum Genet. 2018;102(1):27-43. 10.1016/j.ajhg.2017.10.002 29276006 PMC5777383

[8] Saraç F, Baş A, Çelikel P, Şengül F. The intraoral findings of the patient with Robinow syndrome and the related dental treatment approaches: a case report. J Adv Oral Res. 2023;14(2):218-222. 10.1177/23202068231199538

[9] Zhang C, Jolly A, Shayota BJ, et al. Novel pathogenic variants and quantitative phenotypic analyses of Robinow syndrome: WNT signaling perturbation and phenotypic variability. HGG Adv. 2022;3(1):100074. 10.1016/j.xhgg.2021.100074 35047859 PMC8756549

[10] Zhang C, Mazzeu JF, Eisfeldt J, et al. Novel pathogenic genomic variants leading to autosomal dominant and recessive Robinow syndrome. Am J Med Genet A. 2021;185(12, 12):3593-3600. 10.1002/ajmg.a.61908 33048444 PMC8445516

[11] Yamaguchi TP, Bradley A, McMahon AP, Jones S. A Wnt5a pathway underlies outgrowth of multiple structures in the vertebrate embryo. Development. 1999;126(6):1211-1223. 10.1242/dev.126.6.1211 10021340

[12] Hosseini-Farahabadi S, Gignac S, Danescu A, Fu K, Richman J. Abnormal WNT5A signaling causes mandibular hypoplasia in Robinow syndrome. J Dent Res. 2017;96(11):1265-1272. 10.1177/0022034517716916 28662348

[13] Lin M, Li L, Liu C, et al. Wnt5a regulates growth, patterning, and odontoblast differentiation of developing mouse tooth. Dev Dyn. 2011;240(2):432-440. 10.1002/dvdy.22550 21246660 PMC3023990

[14] Hosseini-Farahabadi S, Geetha-Loganathan P, Fu K, Nimmagadda S, Yang HJ, Richman JM. Dual functions for WNT5A during cartilage development and in disease. Matrix Biol. 2013;32(5):252-264. 10.1016/j.matbio.2013.02.005 23474397

[15] Tokavanich N, Wein MN, English JD, Ono N, Ono W. The role of Wnt signaling in postnatal tooth root development. Front Dent Med. 2021;2:769134. 10.3389/fdmed.2021.769134 35782525 PMC9248717

[16] Xiang L, Chen M, He L, et al. Wnt5a regulates dental follicle stem/progenitor cells of the periodontium. Stem Cell Res Ther. 2014;5(6):1-9. 10.1186/scrt525 PMC4446079 25510849

[17] Sarkar L, Sharpe PT. Expression of Wnt signalling pathway genes during tooth development. Mech Dev. 1999;85(1-2):197-200. 10.1016/S0925-4773(99)00095-7 10415363

[18] Maeda K, Kobayashi Y, Koide M, et al. The regulation of bone metabolism and disorders by Wnt signaling. Int J Mol Sci. 2019;20(22):5525. 10.3390/ijms20225525 31698687 PMC6888566

[19] van Amerongen R, Fuerer C, Mizutani M, Nusse R. Wnt5a can both activate and repress Wnt/β-catenin signaling during mouse embryonic development. Dev Biol. 2012;369(1):101-114. 10.1016/j.ydbio.2012.06.020 22771246 PMC3435145

[20] Maeda K, Kobayashi Y, Udagawa N, et al. Wnt5a-Ror2 signaling between osteoblast-lineage cells and osteoclast precursors enhances osteoclastogenesis. Nat Med. 2012;18(3):405-412. 10.1038/nm.2653 22344299

[21] Houchen CJ, Castro B, Leat PH, Mohammad N, Hall-Glenn F, Bumann EE. Treatment with an inhibitor of matrix metalloproteinase 9 or cathepsin K lengthens embryonic lower jaw bone. Orthod Craniofac Res. 2023;26(3):500-509. 10.1111/ocr.12635 36680416 PMC11508777

[22] Houchen CJ, Ghanem S, Kaartinen V, Bumann EE. TGF-β signaling in the cranial neural crest affects late-stage mandibular bone resorption and length. Front Physiol. 2024;15. 10.3389/fphys.2024.1435594 PMC11519526 39473613

[23] Ealba EL, Jheon AH, Hall J, Curantz C, Butcher KD, Schneider RA. Neural crest-mediated bone resorption is a determinant of species-specific jaw length. Dev Biol. 2015;408(1):151-163. 10.1016/j.ydbio.2015.10.001 26449912 PMC4698309

[24] Hall J, Jheon AH, Ealba EL, et al. Evolution of a developmental mechanism: species-specific regulation of the cell cycle and the timing of events during craniofacial osteogenesis. Dev Biol. 2014;385(2):380-395. 10.1016/j.ydbio.2013.11.011 24262986 PMC3953612

[25] Hassan MG, Vargas R, Zhang B, Marcel N, Cox TC, Jheon AH. Altering osteoclast numbers using CTSK models in utero affects mice offspring craniofacial morphology. Orthod Craniofac Res. 2022;26(3):338-348. 10.1111/ocr.12614 36245435

[26] Chai W, Hao W, Liu J, et al. Visualizing cathepsin K cre expression at the single cell level with GFP reporters. JBMR Plus. 2023;7(1):e10706. 10.1002/jbm4.10706 36699636 PMC9850439

[27] Ding Y, Mo C, Geng J, Li J, Sun Y. Identification of periosteal osteogenic progenitors in jawbone. J Dent Res. 2022;101(9):1101-1109. 10.1177/00220345221084200 35319300

[28] Debnath S, Yallowitz AR, McCormick J, et al. Discovery of a periosteal stem cell mediating intramembranous bone formation. Nature. 2018;562(7725):133-139. 10.1038/s41586-018-0554-8 30250253 PMC6193396

[29] Nakamura T, Imai Y, Matsumoto T, et al. Estrogen prevents bone loss via estrogen receptor α and induction of Fas ligand in osteoclasts. Cell. 2007;130(5):811-823. 10.1016/j.cell.2007.07.025 17803905

[30] Chiu WSM, Mcmanus JF, Notini AJ, Cassady AI, Zajac JD, Davey RA. Transgenic mice that express Cre recombinase in osteoclasts, Genesis. 2004;39(3):178-185. 10.1002/gene.20041 15282744

[31] Roberts JL, Liu G, Paglia DN, et al. Deletion of Wnt5a in osteoclasts results in bone loss through decreased bone formation. Ann N Y Acad Sci. 2020;1463(1):45-59. 10.1111/nyas.14293 31919867

[32] Youlten SE, Kemp JP, Logan JG, et al. Osteocyte transcriptome mapping identifies a molecular landscape controlling skeletal homeostasis and susceptibility to skeletal disease. Nat Commun. 2021;12(1):2444. 10.1038/s41467-021-22517-1 33953184 PMC8100170

[33] Ge X, Shi R, Ma X. The secreted protein WNT5A regulates condylar chondrocyte proliferation, hypertrophy and migration. Arch Oral Biol. 2017;82:171-179. 10.1016/j.archoralbio.2017.06.019 28647646

[34] Gong W, Yue Z, Chu H, Mi X, Li Y. Tensile stress promotes the chondrogenic ability of condylar cartilage stem/progenitor cells in the temporomandibular joint via the Piezo1-Ca2+-Prkca pathway. Stem Cell Res Ther. 2025;16(1):331. 10.1186/s13287-025-04439-7 40598380 PMC12210528

[35] Chen J, Tu X, Esen E, et al. WNT7B promotes bone formation in part through mTORC1. PLoS Genet. 2014;10(1):e1004145. 10.1371/journal.pgen.1004145 24497849 PMC3907335

[36] Vora SR, Camci ED, Cox TC. Postnatal ontogeny of the cranial base and craniofacial skeleton in male C57BL/6J mice: a reference standard for quantitative analysis. Front Physiol. 2016;6:417. 10.3389/fphys.2015.00417 26793119 PMC4709510

[37] Hassan MG, Kaler H, Zhang B, Cox TC, Young N, Jheon AH. Effects of multi-generational soft diet consumption on mouse craniofacial morphology. Front Physiol. 2020;11:783. 10.3389/fphys.2020.00783 32754047 PMC7367031

[38] Bamaga I, O’Sullivan R, Schmitz J, Fath W, Fajardo R. An osteoblast origin for craniofacial dysplasia in neurofibromatosis type I. J Dent Health Oral Disord Ther. 2017;6(6):00223. 10.15406/jdhodt.2017.06.00223

[39] MacLeod N . PalaeoMath: Part 15-Size & Shape Coordinates. Palaeontology Newsletter; 2008:69.

[40] Maga AM, Navarro N, Cunningham ML, Cox TC. Quantitative trait loci affecting the 3D skull shape and size in mouse and prioritization of candidate genes in-silico. Front Physiol. 2015;6. 10.3389/fphys.2015.00092 PMC4374467 25859222

[41] Klingenberg CP . Size, shape, and form: concepts of allometry in geometric morphometrics. Dev Genes Evol. 2016;226(3):113-137. 10.1007/s00427-016-0539-2 27038023 PMC4896994

[42] Rohlf FJ . tpsDig, version 2.10. Accessed March 11, 2025. http://life.bio.sunysb.edu/morph/index html. 2006.

[43] Adams DC, Otárola-Castillo E. geomorph: an R package for the collection and analysis of geometric morphometric shape data. Methods Ecol Evol. 2013;4(4):393-399. 10.1111/2041-210X.12035

[44] Schindelin J, Arganda-Carreras I, Frise E, et al. Fiji: an open-source platform for biological-image analysis. Nat Methods. 2012;9(7):676-682. 10.1038/nmeth.2019 22743772 PMC3855844

[45] Koo TK, Li MY. A guideline of selecting and reporting intraclass correlation coefficients for reliability research. J Chiropr Med. 2016;15(2):155-163. 10.1016/j.jcm.2016.02.012 27330520 PMC4913118

[46] Simonson L, Oldham E, Chang H. Overactive Wnt5a signaling disrupts hair follicle polarity during mouse skin development. Development. 2022;149(22). 10.1242/dev.200816 PMC9845745 36305473

[47] Yang T, Zhang J, Cao Y, et al. Wnt5a/Ror2 mediates temporomandibular joint subchondral bone remodeling. J Dent Res. 2015;94(6):803-812. 10.1177/0022034515576051 25749876 PMC6728679

[48] Minegishi K, Hashimoto M, Ajima R, et al. A Wnt5 activity asymmetry and intercellular signaling via PCP proteins polarize node cells for left-right symmetry breaking. Dev Cell. 2017;40(5):439-452.e4. 10.1016/j.devcel.2017.02.010 28292423 PMC7974384

[49] Vuga LJ, Ben-Yehudah A, Kovkarova-Naumovski E, et al. WNT5A is a regulator of fibroblast proliferation and resistance to apoptosis. Am J Respir Cell Mol Biol. 2009;41(5):583-589. 10.1165/rcmb.2008-0201OC 19251946 PMC2778165

[50] Bo H, Gao L, Chen Y, Zhang J, Zhu M. Upregulation of the expression of Wnt5a promotes the proliferation of pancreatic cancer cells in vitro and in a nude mouse model. Mol Med Rep. 2016;13(2):1163-1171. 10.3892/mmr.2015.4642 26648282 PMC4732830

[51] Zhu S, Huo S, He W, et al. Fine-tuning of Wnt signaling by RNA surveillance factor Smg5 in the mouse craniofacial development. iScience. 2025;28(3):111972. 10.1016/j.isci.2025.111972 40071146 PMC11894330

[52] Shayota BJ, Zhang C, Shypailo RJ, Mazzeu JF, Carvalho CM, Sutton VR. Characterization of the Robinow syndrome skeletal phenotype, bone micro-architecture, and genotype–phenotype correlations with the osteosclerotic form. Am J Med Genet A. 2020;182(11):2632-2640. 10.1002/ajmg.a.6184332888393

