# Supplementary material for: Wnt5a gain- and loss-of-function in bone have distinct craniofacial phenotypes
Source: JBMR Plus. 2026 Apr 7;10(6):ziag060. doi: 10.1093/jbmrpl/ziag060 (PMC13156483; doi:10.1093/jbmrpl/ziag060)
Supplement: Houchen_Wnt5a_Supplement-PUBLICATION_CJH_033026_v11_ziag060 [file houchen_wnt5a_supplement-publication_cjh_033026_v11_ziag060.docx]

**SUPPLEMENTARY MATERIAL**

**Wnt5a gain- and loss-of-function in bone have distinct craniofacial phenotypes**

Claire J. Houchen, Portia Hahn Leat, Cassandra Delich, Jocelyn Vang, Sara Yingling-Haggard, Joseph L. Roberts, Hicham Drissi, Erin E. Bumann

**Contents**

Supplemental Figures..............................2-9

Supplemental Tables..............................10-17

**Supplemental Figures**


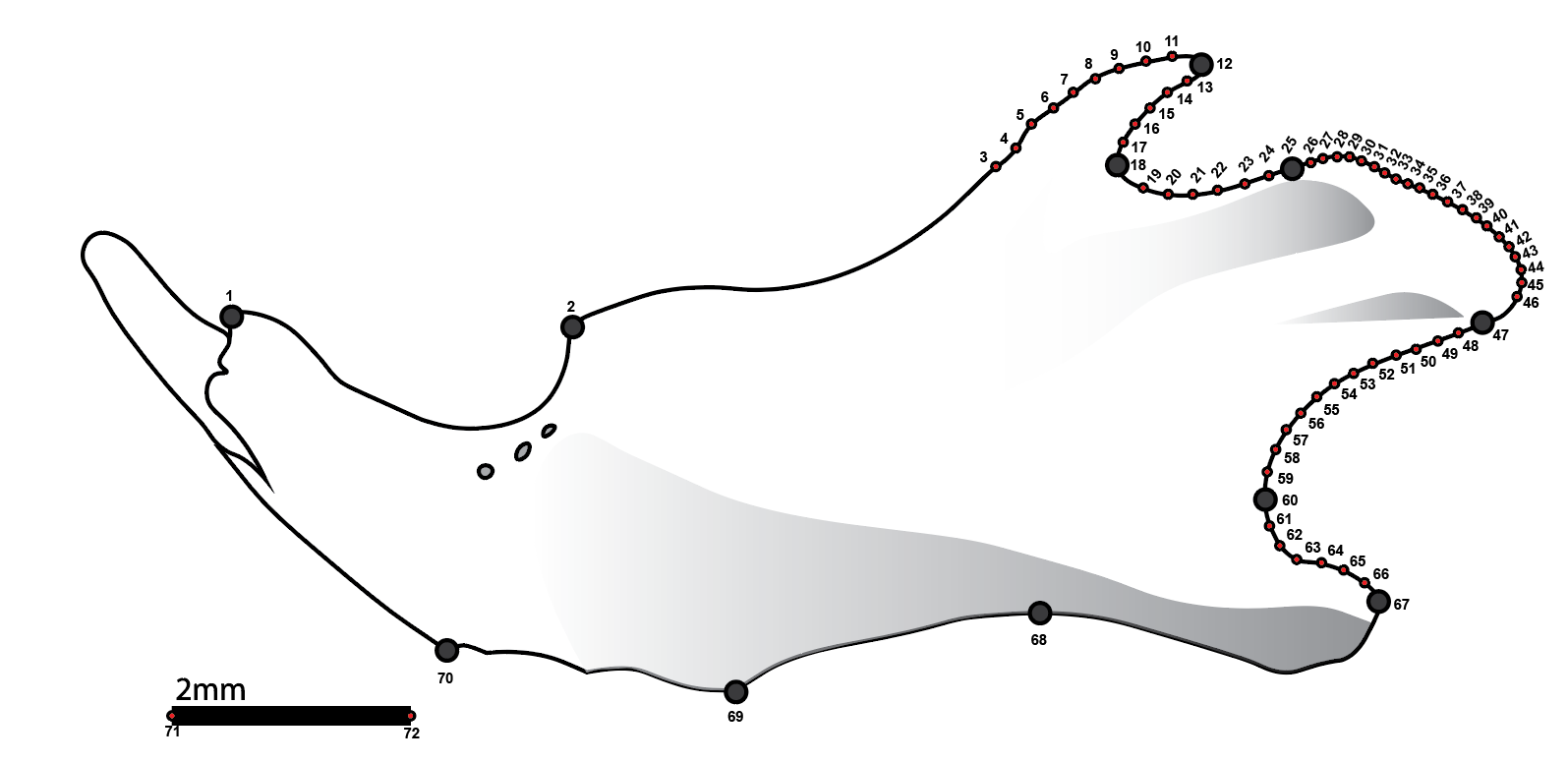


| 3D LM | 2D LM | Type | Landmark Descriptions |
| --- | --- | --- | --- |
| M01 | 1 |  | Most superior point of incisor alveolus (Midpoint on alveolar bone lingual to the mandibular incisor) |
| M02 | 2 |  | Most anterior point of the first molar alveolus |
|  | 3-11 | Semi- | A line was drawn between 2(M02) and 12(M04) and divided into three equal sections. Semi-landmarks 3-11 were placed in the 3rd section, on this line along the anterior curve of the coronoid process. |
| M04 | 12 |  | Most posterior tip of the coronoid process |
|  | 13-17 | Semi- | A line was drawn between 12(M04) and 18(M05). Semi-landmarks 13-17 were placed equidistant on this line along the posterior curve of the coronoid process. |
| M05 | 18 |  | Most anterior/inferior concave point of the coronoid process |
|  | 19-24 | Semi- | A line was drawn between 18(M05) and 25(M06). Semi-landmarks 19-24 were placed equidistant on this line along the dorsal curve of the neck of the condylar process. |
| M06 | 25 |  | Most anterior point of the articular surface of the condyle |
|  | 26-46 | Semi- | A line was drawn between 25(M06) and 47(M07) and divided into two equal sections. Landmark 36 was placed on the center of this line, along the curve of the condylar process. A line was drawn between landmarks 25 and 36, and semi-landmarks were placed equidistant on this line, along the curve of the condylar process. This was then repeated for semi-landmarks placed between 36 and 47 along the curve of the condylar process. |
| M07 | 47 |  | Most posterior tip of the condyle (Posterior inferior point on mandibular condyle) |
|  | 48-59 | Semi- | A line was drawn between 47(M07) and 60(M08). Semi-landmarks 48-59 were placed equidistant on this line along the ventral curve of the neck of the condylar process. |
| M08 | 60 |  | Most anterior concave point between the condyle and the angle of mandible |
|  | 61-66 | Semi- | A line was drawn between 60(M08) and 67(M09). Semi-landmarks 61-66 were placed equidistant on this line along the dorsal curve of the neck of the angular process. |
| M09 | 67 |  | Most posterior tip of the mandibular angle (Posterior tip of the angular process) |
| M11 | 68 |  | Ascending ramus dorsal-most ventral point |
| M12 | 69 |  | Most inferior point of the alveolar region |
| M13 | 70 |  | Anterior inferior most point on the body of the mandible |
|  | 71-72 | Scale Bar | Landmarks are placed at the ends of the scale bar to use for resizing landmarks to correct for size variations in screenshots. |

**Supplemental Figure 1**. Three-dimensionally placed landmarks (large dots) and two-dimensionally placed semi-landmarks (small dots) used for mandible processes geometric morphometric analysis, and description of all mandibular landmarks placed for geometric morphometric analysis of mandible processes shape. LM=landmark

**
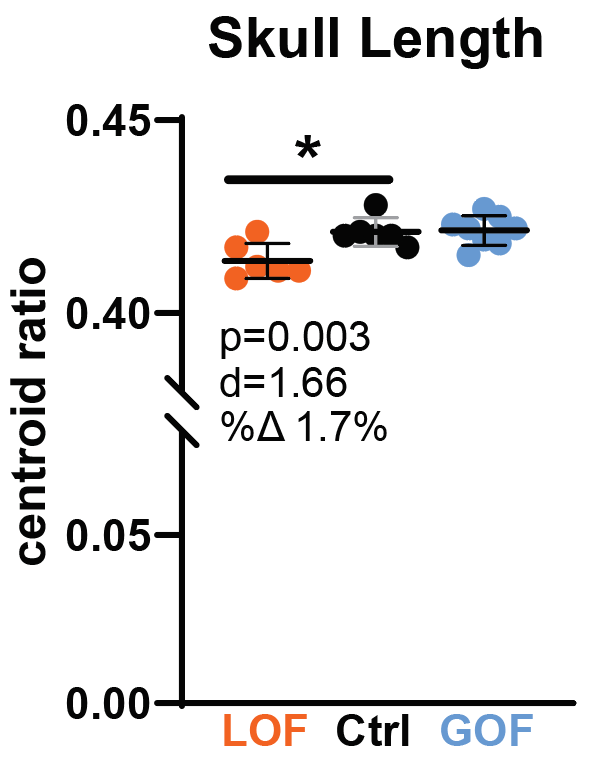
**

**Supplemental Figure 2**. Total skull length was a significant 1.7% smaller in *Wnt5a* LOF mice.


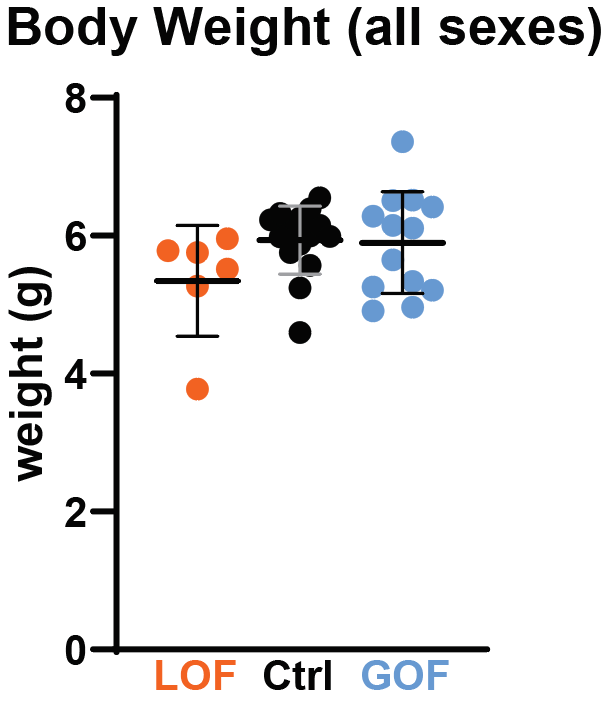


**Supplemental Figure 3**. Body weight at P10 did not differ between groups.


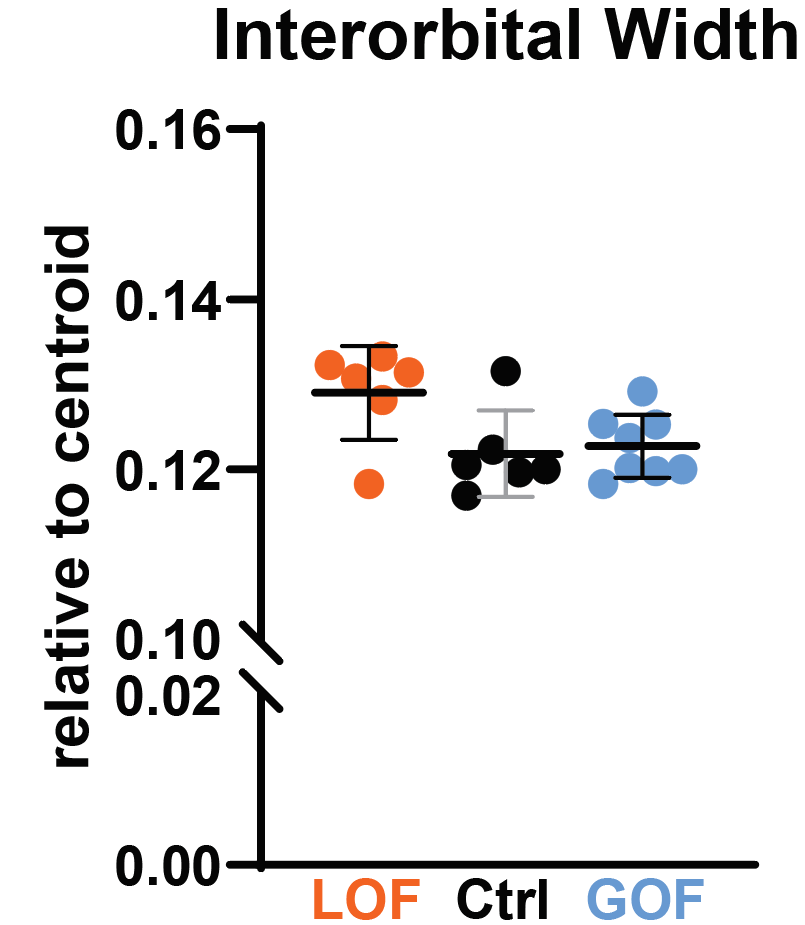


**Supplemental Figure 4**. Interorbital width, indicative of hypertelorism, trended up in *Wnt5a* LOF mice versus controls (p=0.0524) but did not meet the threshold of statistical significance using a false discovery rate (p<.01).


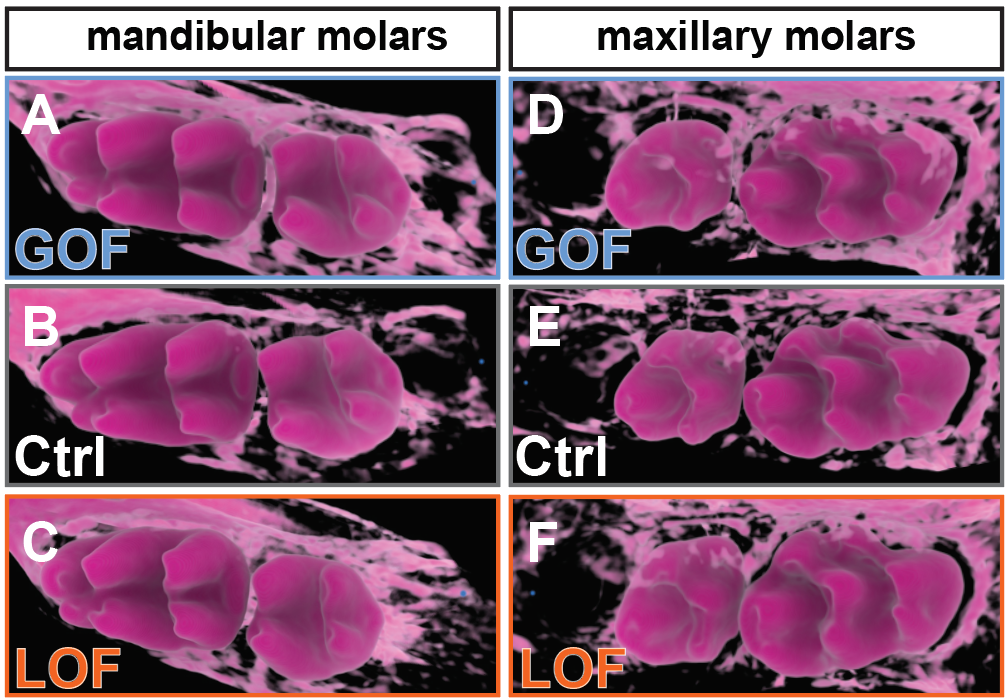


**Supplemental Figure 5**. Severe morphology changes were not observed in the dentition. **A-C)** mandibular and **D-F)** maxillary molars were similar in all genotypes. Images are pseudocolored pink for visualization of morphology, but color does not indicate mineral density.


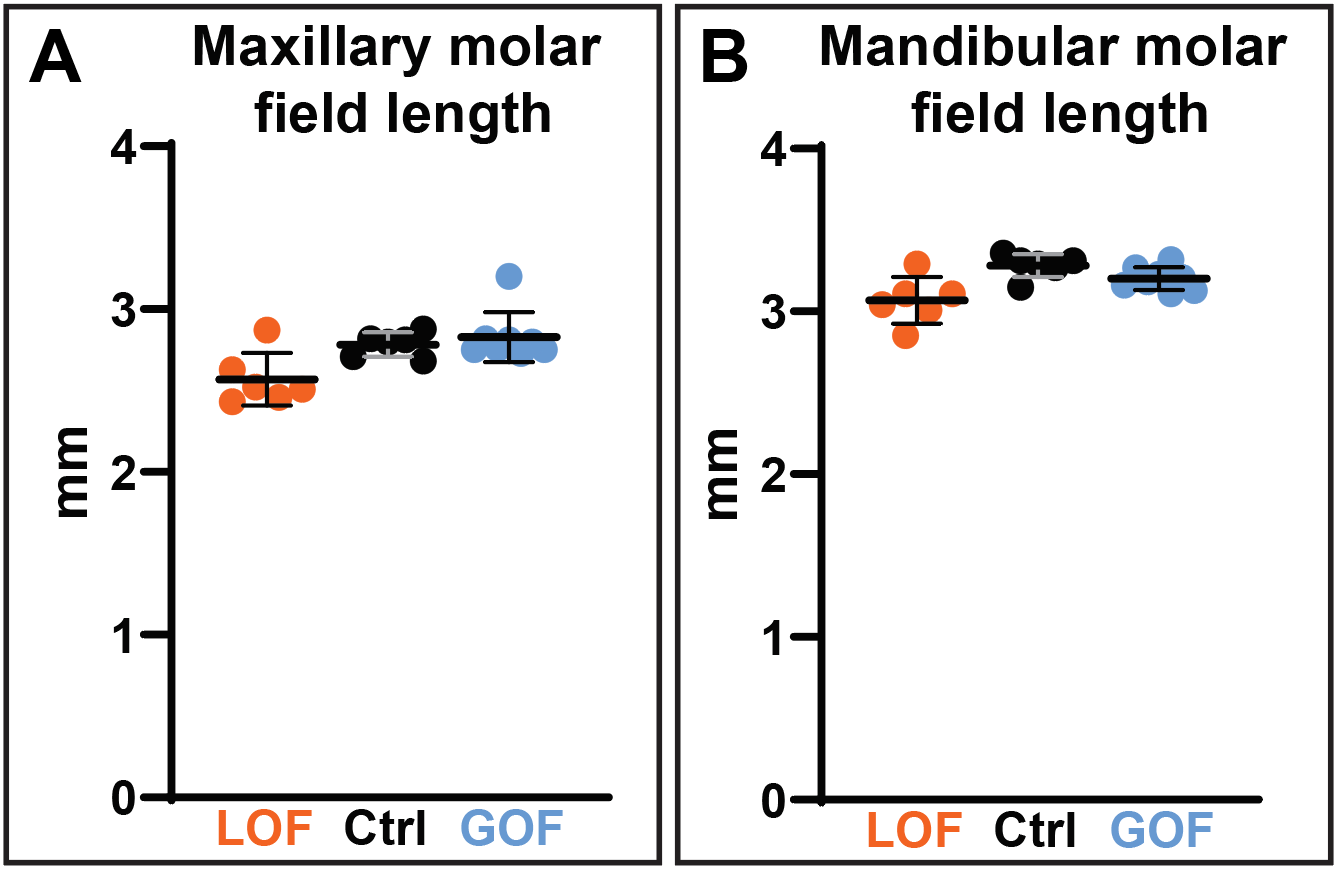


**Supplemental Figure 6**. A) Wnt5a LOF mice had a 7.7% shorter maxillary molar field length (p=0.022) and B) a 6.5% shorter mandibular molar field length (p=0.013) versus controls, but the changes did not meet the threshold of statistical significance using a false discovery rate and p-value cutoff of p<.01.


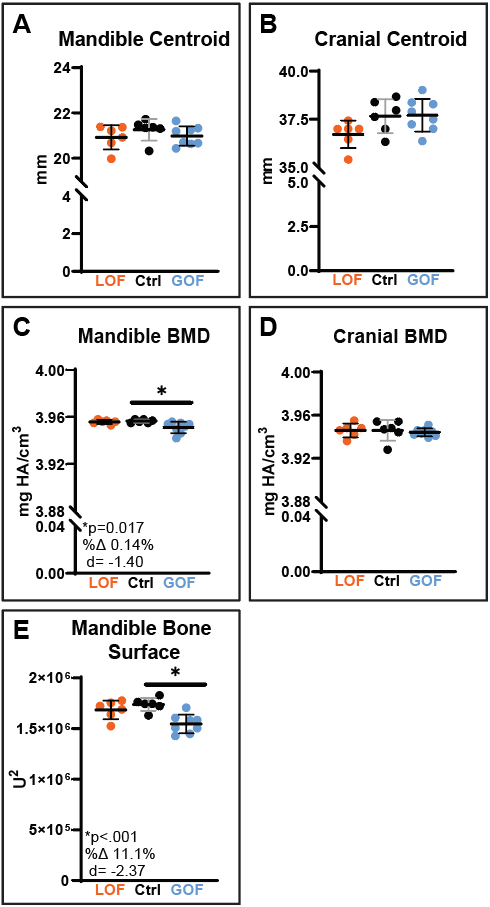


**Supplemental Figure 7**. A) Mandible centroid size did not differ between genotypes, but B) cranial centroid size trended down in *Wnt5a* LOF mice (p=0.067). C) Mandible BMD was significantly decreased in *Wnt5a* GOF mice, but not to a biologically impactful extent (0.14% change). D) Cranial BMD did not differ between groups. E) Mandible bone surface was a significant 11.1% less in *Wnt5a* GOF mice.


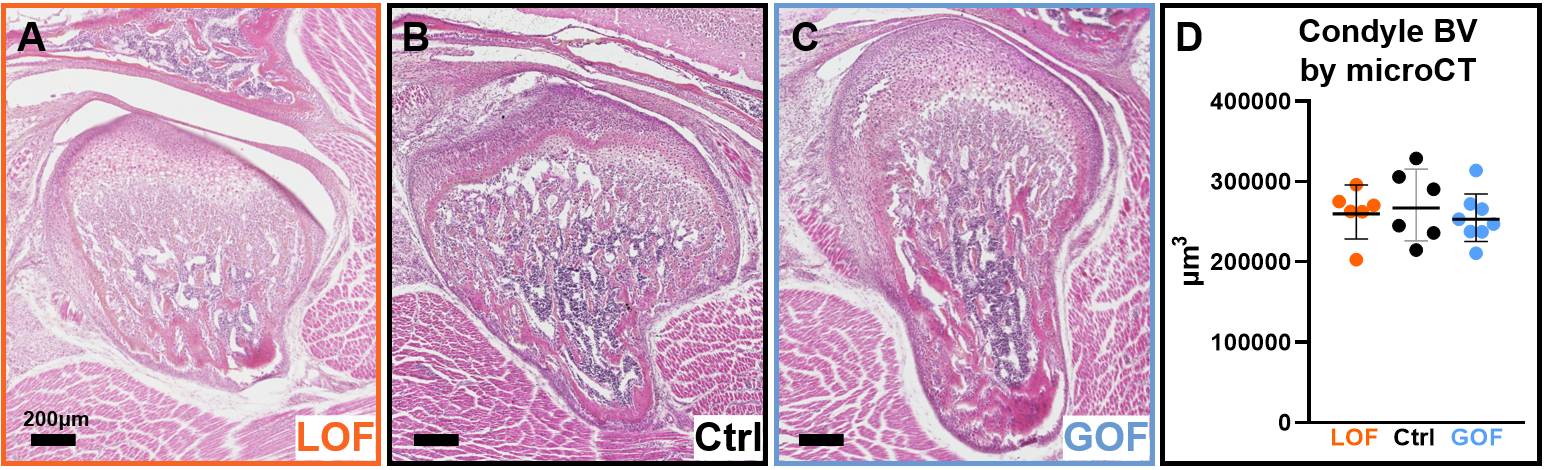


**Supplemental Figure 8**. Representative H&E staining of sagittal sections through the mandibular condyle (TMJ) of A) *Wnt5a* LOF, B) control, and C) GOF mice. D) Mandibular condyle bone volume (BV), measured by microCT, did not significantly differ between genotypes, suggesting the change in Wnt5a GOF condyles is due to dysmorphology rather than underdevelopment.

**Supplemental Tables**

|  |  |  |  |  |  |  |  |  |  | Unpaired t test with Welch correction, FDR (Q)=5 | | | |
| --- | --- | --- | --- | --- | --- | --- | --- | --- | --- | --- | --- | --- | --- |
|  | **Wnt5afl/fl (Ctrl)** | | | **Ctsk-Cre;Wnt5afl/fl (LOF)** | | | **Ctsk-Cre;Rosa26-LSL-Wnt5a(GOF)** | | | **Wnt5afl/fl vs Ctsk-Cre;Wnt5afl/fl (LOF)** | | **Wnt5afl/fl vs Ctsk-Cre;Rosa26-LSL-Wnt5a (GOF)** | |
|  | **Mean** | **StDev** | **N** | **Mean** | **StDev** | **N** | **Mean** | **StDev** | **N** | **P value** | **effect size (d)** | **P value** | **effect size (d)** |
| Basisphenoid (rostral) | 0.0268 | 0.0020 | 6 | 0.0307 | 0.0019 | 6 | 0.0279 | 0.0010 | 8 | **0.0058** | **-2.018** | 0.2516 | NA |
| Basisphenoid (caudal) | 0.0481 | 0.0014 | 6 | 0.0501 | 0.0034 | 6 | 0.0493 | 0.0009 | 8 | 0.2227 | NA | 0.1055 | NA |
| Cranial base (posterior region) | 0.0970 | 0.0027 | 6 | 0.0996 | 0.0026 | 6 | 0.0981 | 0.0016 | 8 | 0.1165 | NA | 0.4131 | NA |
| Inter-mastoid width | 0.2775 | 0.0043 | 6 | 0.2828 | 0.0058 | 6 | 0.2735 | 0.0045 | 8 | 0.1027 | NA | 0.1202 | NA |
| Inter-zygomatic root width | 0.0616 | 0.0035 | 6 | 0.0623 | 0.0038 | 6 | 0.0599 | 0.0065 | 8 | 0.7702 | NA | 0.5430 | NA |
| Anterior cranial vault width | 0.1545 | 0.0069 | 6 | 0.1598 | 0.0073 | 6 | 0.1595 | 0.0040 | 8 | 0.2209 | NA | 0.1535 | NA |
| Interior frontal arch width | 0.0854 | 0.0052 | 6 | 0.0870 | 0.0054 | 6 | 0.0892 | 0.0035 | 8 | 0.5984 | NA | 0.1606 | NA |
| Inter-zygomatic arch width | 0.2013 | 0.0031 | 6 | 0.2068 | 0.0055 | 6 | 0.2061 | 0.0028 | 8 | 0.0654 | NA | 0.0130 | NA |
| Inter-orbital width | 0.1220 | 0.0052 | 6 | 0.1288 | 0.0056 | 6 | 0.1226 | 0.0037 | 8 | 0.0524 | **-1.271** | 0.8073 | NA |
| Anterior nasal width | 0.0613 | 0.0026 | 6 | 0.0639 | 0.0027 | 6 | 0.0599 | 0.0018 | 8 | 0.1288 | NA | 0.2775 | NA |
| Inter-maxillary width | 0.1267 | 0.0026 | 6 | 0.1347 | 0.0060 | 6 | 0.1275 | 0.0018 | 8 | 0.0200 | NA | 0.5151 | NA |
| Inter-molar width | 0.0916 | 0.0039 | 6 | 0.0992 | 0.0027 | 6 | 0.0896 | 0.0031 | 8 | **0.0035** | **-2.270** | 0.3345 | NA |
| Palatal width | 0.0338 | 0.0011 | 6 | 0.0356 | 0.0016 | 6 | 0.0353 | 0.0014 | 8 | 0.0425 | NA | 0.0454 | NA |
| Posterior cranial vault | 0.1327 | 0.0015 | 6 | 0.1357 | 0.0054 | 6 | 0.1376 | 0.0035 | 8 | 0.2417 | NA | **0.0053** | **-1.724** |
| Middle cranial vault | 0.1650 | 0.0032 | 6 | 0.1702 | 0.0055 | 6 | 0.1710 | 0.0040 | 8 | 0.0818 | NA | **0.0094** | **-1.613** |
| Anterior cranial vault | 0.1542 | 0.0035 | 6 | 0.1585 | 0.0051 | 6 | 0.1606 | 0.0030 | 8 | 0.1201 | NA | **0.0046** | **-2.020** |
| Frontal crest height | 0.0998 | 0.0027 | 6 | 0.1046 | 0.0034 | 6 | 0.1061 | 0.0043 | 8 | 0.0249 | NA | **0.0060** | **-1.691** |
| Anterior nasal height | 0.0486 | 0.0011 | 6 | 0.0485 | 0.0030 | 6 | 0.0483 | 0.0022 | 8 | 0.9405 | NA | 0.7596 | NA |
| Facial height | 0.0921 | 0.0029 | 6 | 0.0990 | 0.0091 | 6 | 0.0946 | 0.0044 | 8 | 0.1256 | NA | 0.2328 | NA |
| Posterior nasal height | 0.0262 | 0.0008 | 6 | 0.0276 | 0.0020 | 6 | 0.0256 | 0.0023 | 8 | 0.1743 | NA | 0.4864 | NA |
| Anterior pharyngeal height | 0.0245 | 0.0016 | 6 | 0.0236 | 0.0007 | 6 | 0.0249 | 0.0009 | 8 | 0.2325 | NA | 0.6205 | NA |
| Posterior pharyngeal height | 0.0372 | 0.0013 | 6 | 0.0362 | 0.0017 | 6 | 0.0362 | 0.0019 | 8 | 0.2757 | NA | 0.2893 | NA |
| Ear height | 0.1797 | 0.0049 | 6 | 0.1847 | 0.0061 | 6 | 0.1854 | 0.0055 | 8 | 0.1479 | NA | 0.0643 | NA |
| Palate height | 0.0176 | 0.0010 | 6 | 0.0170 | 0.0015 | 6 | 0.0155 | 0.0036 | 8 | 0.4188 | NA | 0.1478 | NA |

**Supplemental Table 1.** Traditional morphometrics - means, standard deviations, and sample size**.** All measurements relative to centroid size (not raw measurements), except as noted. Statistics imported from GraphPad Prism.

|  |  |  |  |  |  |  |  |  |  | Unpaired t test with Welch correction, FDR (Q)=5 | | | |
| --- | --- | --- | --- | --- | --- | --- | --- | --- | --- | --- | --- | --- | --- |
|  | **Wnt5afl/fl (Ctrl)** | | | **Ctsk-Cre;Wnt5afl/fl (LOF)** | | | **Ctsk-Cre;Rosa26-LSL-Wnt5a(GOF)** | | | **Wnt5afl/fl vs Ctsk-Cre;Wnt5afl/fl (LOF)** | | **Wnt5afl/fl vs Ctsk-Cre;Rosa26-LSL-Wnt5a (GOF)** | |
|  | **Mean** | **StDev** | **N** | **Mean** | **StDev** | **N** | **Mean** | **StDev** | **N** | **P value** | **effect size (d)** | **P value** | **effect size (d)** |
| Total skull length | 0.4220 | 0.0034 | 6 | 0.4148 | 0.0051 | 6 | 0.4221 | 0.0041 | 8 | 0.0190 | **1.658** | 0.9515 | NA |
| Cranial vault length | 0.3090 | 0.0035 | 6 | 0.3155 | 0.0045 | 6 | 0.3093 | 0.0030 | 8 | 0.0203 | NA | 0.8911 | NA |
| Cranial base (rostral) | 0.1957 | 0.0016 | 6 | 0.1975 | 0.0032 | 6 | 0.1894 | 0.0023 | 8 | 0.2503 | NA | **0.0001** | **3.107** |
| Anterior cranial base | 0.0735 | 0.0028 | 6 | 0.0778 | 0.0018 | 6 | 0.0759 | 0.0025 | 8 | 0.0124 | NA | 0.1271 | NA |
| Basiocciput | 0.0794 | 0.0023 | 6 | 0.0801 | 0.0031 | 6 | 0.0760 | 0.0016 | 8 | 0.6623 | NA | 0.0119 | NA |
| Basisphenoid | 0.0566 | 0.0014 | 6 | 0.0573 | 0.0022 | 6 | 0.0568 | 0.0013 | 8 | 0.5111 | NA | 0.7868 | NA |
| Presphenoid | 0.0680 | 0.0011 | 6 | 0.0687 | 0.0010 | 6 | 0.0664 | 0.0010 | 8 | 0.2750 | NA | 0.0191 | NA |
| Facial region length | 0.2050 | 0.0046 | 6 | 0.1952 | 0.0074 | 6 | 0.2013 | 0.0038 | 8 | 0.0230 | NA | 0.1380 | NA |
| Palate length | 0.0447 | 0.0013 | 6 | 0.0443 | 0.0012 | 6 | 0.0421 | 0.0016 | 8 | 0.5267 | NA | **0.0052** | **1.786** |
| Maxilla length | 0.0923 | 0.0037 | 6 | 0.0834 | 0.0044 | 6 | 0.0878 | 0.0019 | 8 | **0.0037** | **2.185** | 0.0320 | NA |
| Premaxilla length | 0.0790 | 0.0016 | 6 | 0.0779 | 0.0027 | 6 | 0.0810 | 0.0030 | 8 | 0.4165 | NA | 0.1362 | NA |
| Nasal length | 0.1052 | 0.0050 | 6 | 0.1027 | 0.0027 | 6 | 0.1090 | 0.0024 | 8 | 0.3162 | NA | 0.1254 | NA |
| Zygomatic length | 0.0793 | 0.0019 | 6 | 0.0771 | 0.0049 | 6 | 0.0823 | 0.0028 | 8 | 0.3418 | NA | 0.0323 | NA |
| Upper Jaw length | 0.2717 | 0.0023 | 6 | 0.2603 | 0.0054 | 6 | 0.2703 | 0.0056 | 8 | **0.0023** | **2.727** | 0.5354 | NA |
| Total skull length projected | 0.4210 | 0.0037 | 6 | 0.4135 | 0.0045 | 6 | 0.4214 | 0.0039 | 8 | 0.0111 | NA | 0.8574 | NA |
| Cranial vault length projected | 0.2977 | 0.0071 | 6 | 0.3023 | 0.0061 | 6 | 0.2981 | 0.0038 | 8 | 0.2500 | NA | 0.8903 | NA |
| Cranial base (rostral) projected | 0.1783 | 0.0079 | 6 | 0.1827 | 0.0033 | 6 | 0.1768 | 0.0045 | 8 | 0.2583 | NA | 0.6736 | NA |
| Anterior cranial base projected | 0.0452 | 0.0083 | 6 | 0.0446 | 0.0047 | 6 | 0.0427 | 0.0034 | 8 | 0.8913 | NA | 0.5092 | NA |
| Basiocciput projected | 0.0738 | 0.0039 | 6 | 0.0747 | 0.0020 | 6 | 0.0713 | 0.0010 | 8 | 0.6103 | NA | 0.1845 | NA |
| Basisphenoid projected | 0.0537 | 0.0027 | 6 | 0.0542 | 0.0034 | 6 | 0.0539 | 0.0025 | 8 | 0.7533 | NA | 0.8405 | NA |
| Presphenoid projected | 0.0637 | 0.0026 | 6 | 0.0645 | 0.0015 | 6 | 0.0626 | 0.0014 | 8 | 0.5425 | NA | 0.3880 | NA |
| Facial region length projected | 0.2047 | 0.0055 | 6 | 0.1950 | 0.0074 | 6 | 0.2009 | 0.0040 | 8 | 0.0300 | NA | 0.1894 | NA |
| Palate projected | 0.0399 | 0.0031 | 6 | 0.0406 | 0.0023 | 6 | 0.0382 | 0.0023 | 8 | 0.6506 | NA | 0.2912 | NA |
| Maxilla projected | 0.0901 | 0.0038 | 6 | 0.0807 | 0.0040 | 6 | 0.0851 | 0.0023 | 8 | **0.0020** | **2.397** | 0.0236 | NA |
| Premaxilla projected | 0.0750 | 0.0022 | 6 | 0.0739 | 0.0027 | 6 | 0.0777 | 0.0033 | 8 | 0.4455 | NA | 0.1000 | NA |

**Supplemental Table 1 (continued).** Traditional morphometrics - means, standard deviations, and sample size**.** All measurements relative to centroid size (not raw measurements), except as noted. Statistics imported from GraphPad Prism.

|  |  |  |  |  |  |  |  |  |  | Unpaired t test with Welch correction, FDR (Q)=5 | | | |
| --- | --- | --- | --- | --- | --- | --- | --- | --- | --- | --- | --- | --- | --- |
|  | **Wnt5afl/fl (Ctrl)** | | | **Ctsk-Cre;Wnt5afl/fl (LOF)** | | | **Ctsk-Cre;Rosa26-LSL-Wnt5a(GOF)** | | | **Wnt5afl/fl vs Ctsk-Cre;Wnt5afl/fl (LOF)** | | **Wnt5afl/fl vs Ctsk-Cre;Rosa26-LSL-Wnt5a (GOF)** | |
|  | **Mean** | **StDev** | **N** | **Mean** | **StDev** | **N** | **Mean** | **StDev** | **N** | **P value** | **effect size (d)** | **P value** | **effect size (d)** |
| Nasal projected | 0.0967 | 0.0047 | 6 | 0.0908 | 0.0061 | 6 | 0.0996 | 0.0034 | 8 | 0.0907 | NA | 0.2268 | NA |
| Zygomatic projected | 0.0755 | 0.0015 | 6 | 0.0742 | 0.0045 | 6 | 0.0796 | 0.0029 | 8 | 0.5295 | NA | **0.0060** | **-1.677** |
| Upper Jaw projected | 0.2432 | 0.0035 | 6 | 0.2310 | 0.0060 | 6 | 0.2400 | 0.0062 | 8 | **0.0027** | **2.459** | 0.2541 | NA |
| Mandibular posterior height | 0.0551 | 0.0014 | 6 | 0.0552 | 0.0020 | 6 | 0.0553 | 0.0023 | 8 | 0.9354 | NA | 0.8149 | NA |
| Mandibular length (superior) | 0.2317 | 0.0023 | 6 | 0.2305 | 0.0021 | 6 | 0.2235 | 0.0015 | 8 | 0.3823 | NA | **0.0001** | **4.298** |
| Mandibular length (inferior) | 0.1745 | 0.0010 | 6 | 0.1733 | 0.0031 | 6 | 0.1669 | 0.0014 | 8 | 0.4124 | NA | **<0.000001** | **6.162** |
| Inter-molar width (mandible) | 0.0835 | 0.0033 | 6 | 0.0841 | 0.0033 | 6 | 0.0880 | 0.0026 | 8 | 0.7476 | NA | 0.0231 | NA |
| Bi-condylar width | 0.2247 | 0.0039 | 6 | 0.2317 | 0.0039 | 6 | 0.2244 | 0.0030 | 8 | 0.0116 | NA | 0.8831 | NA |
| Bi-gonial width | 0.1868 | 0.0044 | 6 | 0.1930 | 0.0044 | 6 | 0.1950 | 0.0027 | 8 | 0.0368 | NA | **0.0043** | **-2.303** |
| Mandibular anterior height | 0.0413 | 0.0085 | 6 | 0.0346 | 0.0028 | 6 | 0.0393 | 0.0013 | 8 | 0.1154 | NA | 0.5908 | NA |
| Condylar width | 0.0393 | 0.0027 | 6 | 0.0405 | 0.0015 | 6 | 0.0410 | 0.0011 | 8 | 0.3512 | NA | 0.1814 | NA |
| Angular Process superior length | 0.0292 | 0.0013 | 6 | 0.0308 | 0.0017 | 6 | 0.0296 | 0.0007 | 8 | 0.0971 | NA | 0.5456 | NA |
| Angular Process inferior length | 0.0578 | 0.0033 | 6 | 0.0537 | 0.0036 | 6 | 0.0509 | 0.0035 | 8 | 0.0643 | NA | **0.0028** | **2.049** |
| coronoid-condylar inlet | 0.0003 | 0.0000 | 6 | 0.0003 | 0.0001 | 6 | 0.0002 | 0.0000 | 8 | 0.0817 | NA | **0.0007** | **2.661** |
| posterior mandibular inlet | 0.0007 | 0.0000 | 6 | 0.0008 | 0.0000 | 6 | 0.0007 | 0.0000 | 8 | 0.4880 | NA | 0.0176 | NA |
| ventral mandibular inlet | 0.0006 | 0.0000 | 6 | 0.0007 | 0.0000 | 6 | 0.0007 | 0.0000 | 8 | 0.0816 | NA | **0.0004** | **-2.873** |
| inter-ear width upper | 0.2244 | 0.0031 | 6 | 0.2308 | 0.0024 | 6 | 0.2288 | 0.0037 | 8 | **0.0027** | **-2.322** | 0.0328 | NA |
| inter-ear width lower | 0.1703 | 0.0117 | 6 | 0.1824 | 0.0049 | 6 | 0.1684 | 0.0116 | 8 | 0.0546 | NA | 0.7691 | NA |
| inter-ear width average | 0.1973 | 0.0063 | 6 | 0.2066 | 0.0025 | 6 | 0.1986 | 0.0060 | 8 | 0.0138 | NA | 0.7199 | NA |
| RAW Maxillary molar field length | 2.7833 | 0.0745 | 6 | 2.5700 | 0.1621 | 6 | 2.8275 | 0.1540 | 8 | 0.0220 | **1.691** | 0.4940 | NA |
| RAW Mandibular molar field length | 3.2817 | 0.0711 | 6 | 3.0683 | 0.1446 | 6 | 3.2013 | 0.0710 | 8 | 0.0134 | **1.873** | 0.0603 | NA |

**Supplemental Table 1 (continued).** Traditional morphometrics - means, standard deviations, and sample size**.** All measurements relative to centroid size (not raw measurements), except as noted. Statistics imported from GraphPad Prism.

| **Measurement** | **%CV** | **Type** |  | **Measurement** | **%CV** | **Type** |
| --- | --- | --- | --- | --- | --- | --- |
| Basisphenoid (rostral) | 1.64 | linear |  | UpperJaw R back | 0.78 | linear |
| Basisphenoid (caudal) | 2.21 | linear |  | UpperJaw R Avg | 0.73 | linear |
| Cranial base (posteriorregion) | 0.32 | linear |  | UpperJaw L front | 0.84 | linear |
| Inter-mastoidwidth | 0.30 | linear |  | UpperJaw L back | 0.84 | linear |
| Inter-zygomatic root width R | 0.59 | linear |  | UpperJaw L Avg | 0.84 | linear |
| Inter-zygomatic root width L | 0.34 | linear |  | UpperJaw Avg | 0.78 | linear |
| Inter-zygomatic root width Avg | 0.47 | linear |  | Mandibular posterior height R | 0.62 | linear |
| Anterior cranial vault width | 0.37 | linear |  | Mandibular posterior height L | 0.36 | linear |
| Inter-ear width R | 0.42 | linear |  | Mandibular posterior height Avg | 0.49 | linear |
| Inter-ear width L | 4.14 | linear |  | Mandibularlength (superior) R | 0.12 | linear |
| Inter-ear width Avg | 2.01 | linear |  | Mandibularlength (superior) L | 0.00 | linear |
| Interior frontal arch width | 3.36 | linear |  | Mandibularlength (superior) Avg | 0.06 | linear |
| Inter-zygomatic arch width | 0.55 | linear |  | Mandibularlength (inferior) R | 0.05 | linear |
| Inter-orbital width | 0.42 | linear |  | Mandibularlength (inferior) L | 0.01 | linear |
| Anterior nasal width | 0.38 | linear |  | Mandibularlength (inferior) Avg | 0.02 | linear |
| Inter-maxillary width | 1.33 | linear |  | Mandibular inter-molar width | 2.08 | linear |
| Inter-molar width | 0.02 | linear |  | Bi-condylar width | 0.29 | linear |
| Palatal width | 0.07 | linear |  | Bi-gonial width | 0.03 | linear |
| Posterior cranial vault | 0.01 | linear |  | Mandibular anterior height R | 0.07 | linear |
| Middle cranial vault | 0.02 | linear |  | Mandibular anterior height L | 1.86 | linear |
| Anterior cranial vault | 0.30 | linear |  | Mandibular anterior height Avg | 0.95 | linear |
| Frontal crest height | 0.14 | linear |  | Condylar width R | 2.62 | linear |
| Anterior nasal height | 0.80 | linear |  | Condylar width L | 2.94 | linear |
| Facial height | 0.13 | linear |  | Condylar width Avg | 2.78 | linear |
| Posterior nasal height | 0.15 | linear |  | Angular Process superior length R | 0.92 | linear |
| Anterior pharyngeal height | 1.88 | linear |  | Angular Process superior length L | 1.54 | linear |
| Posterior pharyngeal height R | 0.01 | linear |  | Angular Process superior length Avg | 0.31 | linear |
| Posterior pharyngeal height L | 0.26 | linear |  | Angular Process inferior length R | 2.43 | linear |
| Posterior pharyngeal height Avg | 0.13 | linear |  | Angular Process inferior length L | 3.05 | linear |
| Ear height R up | 0.74 | linear |  | Angular Process inferior length Avg | 2.74 | linear |
| Ear height R down | 0.90 | linear |  | Coronoid-Condylar inlet R | 3.50 | angular |
| Ear height R Avg | 0.83 | linear |  | Coronoid-Condylar inlet L | 6.09 | angular |
| Ear height L up | 0.54 | linear |  | Coronoid-Condylar inlet Avg | 4.77 | angular |
| Ear height L down | 0.56 | linear |  | Posterior mandibular inlet R | 0.36 | angular |
| Ear height L Avg | 0.09 | linear |  | Posterior mandibular inlet L | 1.94 | angular |
| Ear height Avg | 0.46 | linear |  | Posterior mandibular inlet Avg | 1.16 | angular |
| Total skull length | 0.19 | linear |  | Ventral mandibular inlet R | 1.69 | angular |
| Cranial vault length | 0.24 | linear |  | Ventral mandibular inlet L | 0.19 | angular |
| Cranial base (rostral) | 0.02 | linear |  | Ventral mandibular inlet Avg | 0.73 | angular |
| Anterior cranial base | 0.21 | linear |  | Cranial base angle | 0.09 | angular |
| Basiocciput | 0.09 | linear |  | Anterior cranial vault angle | 0.15 | angular |
| Basisphenoid | 0.52 | linear |  | Mid-anterior cranial vault angle | 0.43 | angular |
| Presphenoid | 0.16 | linear |  | Mid-posterior cranial vault angle | 0.15 | angular |
| Facial region length | 0.12 | linear |  | Posterior cranial vault angle | 0.14 | angular |
| Palate | 0.04 | linear |  | Snout angle | 1.69 | angular |
| Maxilla R | 0.23 | linear |  | Facial angle | 0.29 | angular |
| Maxilla L | 0.13 | linear |  | Palatte arch angle | 0.40 | angular |
| Maxilla Avg | 0.05 | linear |  | Nasale-Lambda midline nasal deviation | 0.87 | angular |
| Premaxilla R | 0.56 | linear |  | Bregma-Lambda midline nasal deviation | 1.00 | angular |
| Premaxilla L | 0.13 | linear |  | Face tipping angle | 0.13 | angular |
| Premaxilla Avg | 0.35 | linear |  | Mid-saggital plane midline nasal deviation | 9.32 | angular |
| Nasal | 1.51 | linear |  | Cranial Centroid Size (mm) | 0.09 | neither |
| Zygomatic R | 0.31 | linear |  | Mandibular Centroid Size (mm) | 0.16 | neither |
| Zygomatic L | 0.38 | linear |  | L-Hemimandible Centroid Size (mm) | 0.18 | neither |
| Zygomatic Avg | 0.35 | linear |  | R-Hemimandible Centroid Size (mm) | 0.14 | neither |
| UpperJaw R front | 0.66 | linear |  |  |  |  |

**Supplemental Table 2**. Intraobserver percent coefficient of variation (CV) values. As is standard, a percent CV value of ≤10% was deemed acceptable replicability. R=right; L=left, Avg=average

| Principal Component | Eigenvalues | Proportion of Variance | Cumulative Proportion |
| --- | --- | --- | --- |
| PC1 | 38.134 | 27.238 | 27.238 |
| PC2 | 24.800 | 17.714 | 44.952 |
| PC3 | 18.420 | 13.157 | 58.109 |
| PC4 | 14.020 | 10.014 | 68.124 |
| PC5 | 6.643 | 4.745 | 72.869 |
| PC6 | 5.612 | 4.008 | 76.877 |
| PC7 | 4.603 | 3.288 | 80.165 |
| PC8 | 4.198 | 2.998 | 83.164 |
| PC9 | 3.081 | 2.201 | 85.364 |
| PC10 | 2.346 | 1.675 | 87.040 |
| PC11 | 2.115 | 1.511 | 88.550 |
| PC12 | 1.702 | 1.216 | 89.766 |
| PC13 | 1.549 | 1.106 | 90.872 |
| PC14 | 1.419 | 1.013 | 91.886 |
| PC15 | 1.264 | 0.903 | 92.788 |
| PC16 | 1.083 | 0.773 | 93.562 |
| PC17 | 0.985 | 0.703 | 94.265 |
| PC18 | 0.841 | 0.601 | 94.866 |
| PC19 | 0.819 | 0.585 | 95.451 |
| PC20 | 0.745 | 0.532 | 95.983 |
| PC21 | 0.668 | 0.477 | 96.460 |
| PC22 | 0.621 | 0.444 | 96.904 |
| PC23 | 0.567 | 0.405 | 97.309 |
| PC24 | 0.553 | 0.395 | 97.704 |
| PC25 | 0.470 | 0.336 | 98.040 |
| PC26 | 0.415 | 0.297 | 98.337 |
| PC27 | 0.332 | 0.237 | 98.574 |
| PC28 | 0.293 | 0.209 | 98.783 |
| PC29 | 0.250 | 0.179 | 98.962 |
| PC30 | 0.227 | 0.162 | 99.123 |
| PC31 | 0.194 | 0.139 | 99.262 |
| PC32 | 0.186 | 0.133 | 99.395 |
| PC33 | 0.172 | 0.123 | 99.518 |
| PC34 | 0.168 | 0.120 | 99.638 |
| PC35 | 0.142 | 0.102 | 99.740 |
| PC36 | 0.122 | 0.087 | 99.827 |
| PC37 | 0.085 | 0.061 | 99.888 |
| PC38 | 0.080 | 0.057 | 99.945 |
| PC39 | 0.077 | 0.055 | 100.000 |

**Supplemental Table 3**. Full list of principal components resulting from the geometric morphometric analysis of mandibular processes. PCs 1-4 accounted for >10% of variation.

| ***2x exaggerated*** | **Minimum Component Shape** | **Maximum Component Shape** |
| --- | --- | --- |
| PC1  Proportion of Variance=  **27.23%**  Cumulative Proportion=  **27.23%**  Eigenvalue=  **38.13** | 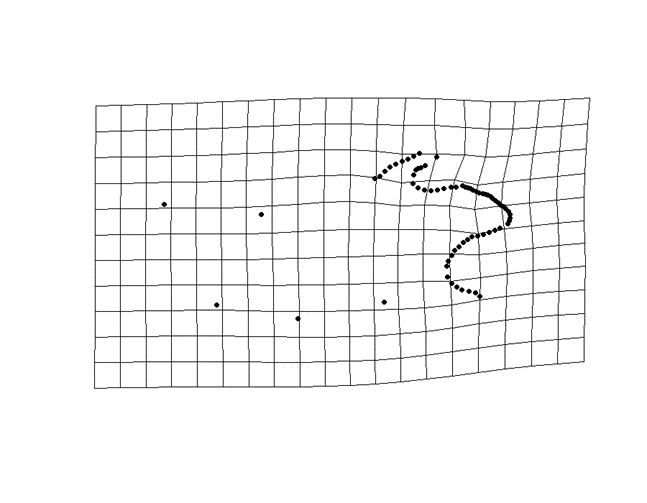 | 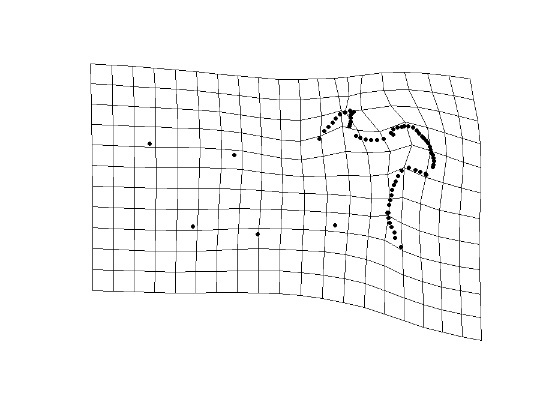 |
| PC2  Proportion of Variance=  **17.71%**  Cumulative Proportion=  **44.95%**  Eigenvalue=  **24.80** | 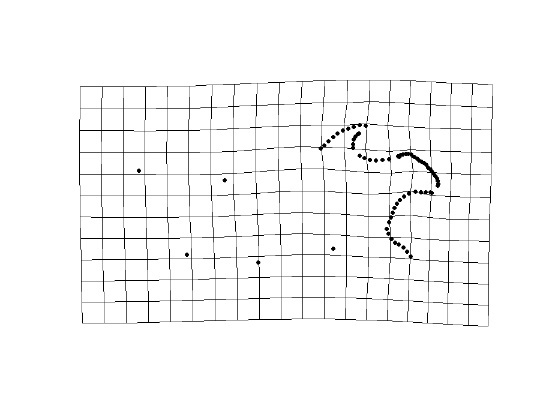 | 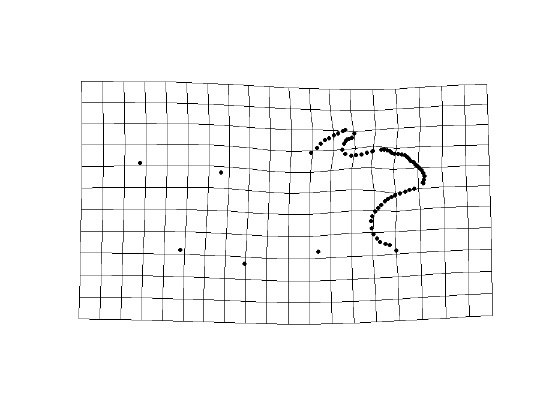 |
| PC3  Proportion of Variance=  **13.16%**  Cumulative Proportion=  **58.11%**  Eigenvalue=  **18.4** | 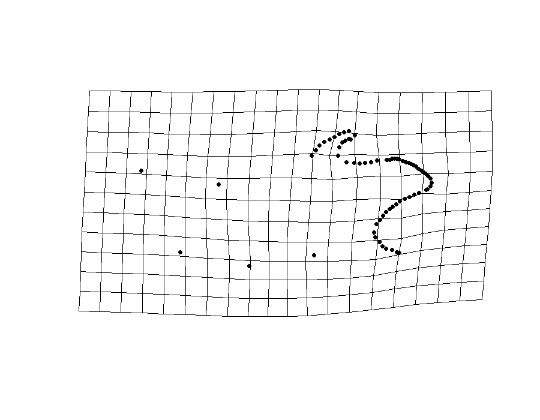 | 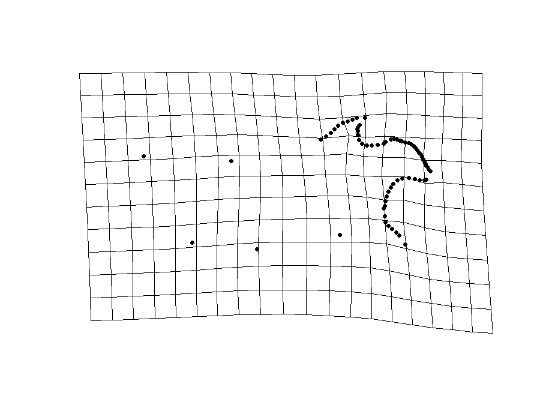 |
| PC4  Proportion of Variance=  **10.01%**  Cumulative Proportion=  **68.12%**  Eigenvalue=  **14.02** | 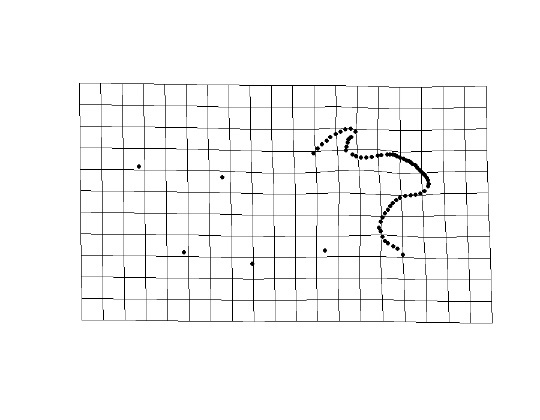 | 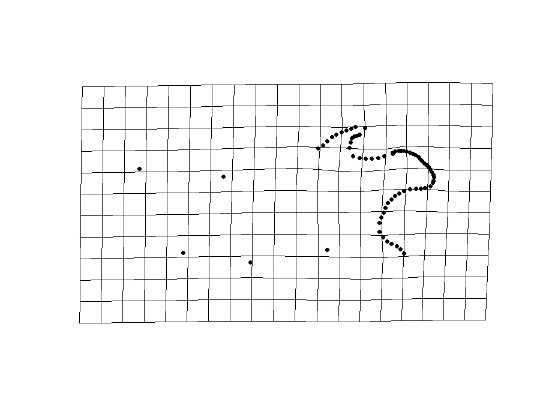 |

**Supplemental Table 4**. PC1-4 were the only PCs describing >10% of variation in the geometric morphometric analysis of mandible processes. Minimum and maximum component shape are shown with 2x exaggeration for each of these PCs.

| **MANCOVA Null Model** | | | | | | | | **Pairwise Comparison** | | | | |
| --- | --- | --- | --- | --- | --- | --- | --- | --- | --- | --- | --- | --- |
|  | **df** | **SS** | **MS** | **Rsq** | **F** | **Z** | **Pr(>F)** |  | **d** | **UCL (95%)** | **Z** | **Pr > d** |
| Centroid | 1 | 119.2 | 119.2 | 0.032 | 1.836 | 1.12 | 0.132 |  |  |  |  |  |
| Genotype | 2 | 1147.7 | 573.84 | 0.309 | 8.839 | 5.754 | 0.0001 | Ctrl vs GOF | 0.029 | 0.017 | 3.658 | 0.0001 |
| Residuals | 36 | 2337 | 64.92 | 0.628 |  |  |  | Ctrl vs LOF | 0.017 | 0.016 | 2.021 | 0.021 |
| Total | 39 | 3719.6 |  |  |  |  |  | GOF vs LOF | 0.03 | 0.016 | 4.218 | 0.0001 |
| **MANCOVA Interaction Model** | | | | | | | | **Coefficients Table** | | | | |
|  | **df** | **SS** | **MS** | **Rsq** | **F** | **Z** | **Pr(>F)** |  | **d.obs** | **UCL (95%)** | **Zd** | **Pr(>d)** |
| Centroid | 1 | 76.7 | 76.717 | 0.021 | 1.299 | 0.626 | 0.269 | Centroid | 63.6 | 83.135 | 0.787 | 0.2244 |
| Genotype | 2 | 329.9 | 164.931 | 0.089 | 2.792 | 2.235 | 0.011 | Genotype GOF | 1485.511 | 1042.794 | 2.96 | 0.0009 |
| Centroid:Genotype | 2 | 328.5 | 164.259 | 0.088 | 2.781 | 2.226 | 0.012 | Genotype LOF | 332.479 | 799.348 | -.712 | 0.7583 |
| Residuals | 34 | 2008.5 | 59.074 | 0.54 |  |  |  | Centroid:Genotype GOF | 242.796 | 170.614 | 2.961 | 0.0009 |
| Total | 39 | 3719.6 |  |  |  |  |  | Centroid:Genotype LOF | 54.157 | 130.131 | -.714 | 0.7582 |

**Supplemental Table 5**. Statistics relating to geometric morphometric analysis of mandibular processes. MANCOVA Null Model: The effects of size and genotype are looked at separately in this model to see if they explain the overall shape variation in the first four principal components (PCs representing >10% of variation). Genotype significantly influences mandibular process morphology and more heavily influences mandibular condyle shape than centroid size. Pairwise Comparison: That genotype influences mandibular condyle shape is driven by differences between all three genotypes. MANCOVA Interaction Model: The effects of size and genotype are looked at together in this model. As in the Null Model, genotype but not centroid significantly influence mandibular process morphology. Additionally, there is a significant interaction of centroid and genotype indicating mandible size correlates with mandibular process morphology within a genotype (allometry). Coefficients Table: This allometric relationship between mandible size and mandibular process morphology occurs in the Wnt5a GOF compared to controls but not Wnt5a LOF mice compared to controls.

| **Summary** | | ***Wnt5a* LOF** *Wnt5a-flox; Ctsk-cre* | ***Wnt5a* GOF** *Rosa26-LSL-Wnt5a;Ctsk-cre* | **Correlating reported clinical phenotype** |
| --- | --- | --- | --- | --- |
|  | P10 body weight (this paper) | --- | --- |  |
|  | P70 body weight^31^ | *↓* males only ^31^ | not assessed |  |
| **P10 craniofacial bone**  (this paper) | maxilla length | **↓** | --- | midface hypoplasia |
|  | upper jaw length | **↓** | --- | midface hypoplasia |
|  | maxillary intermolar width | **↑** | --- |  |
|  | rostral basisphenoid width | **↑** | --- |  |
|  | tooth eruption | delayed | --- | *** |
|  | snout deviation | right sided | right sided | *** |
|  | mandible length | --- | **↓** | micrognathia |
|  | mandible bone surface | --- | **↓** | micrognathia |
|  | mandibular angular process length | --- | **↓** |  |
|  | coronoid and condylar processes morphology | --- | morphology ∆ | *** |
|  | cranial vault height | --- | **↑** | macrocephaly |
|  | palate length | --- | **↓** | short hard palate |
|  | zygomatic length | --- | **↑** |  |
|  | mandible bone surface | --- | **↓** | micrognathia |
| **P70 long bone^31^** | femur BMD | **↓** | not assessed | non-cranial osteopenia |
|  | femur bone surface | **↓** | not assessed |  |

**Supplemental Table 6**. Summary of key findings. Up or down arrow indicates statistically significant increase or decreased in the measure in the indicated group compared to controls, dash indicates no difference. *** should be evaluated in patients with Robinow Syndrome.
